# Understanding the Role of Macrocycle Size and Amide Linkage in Teixobactin Analogues

**DOI:** 10.3390/biom16070970

**Published:** 2026-07-01

**Authors:** Ruba Malkawi, James Weldon-Bee, Edwin Kiptoo, Sanjit Das, Yinzhe Chen, Abhishek Iyer, Rajamani Lakshminarayanan, Qian Zhang, Anish Parmar, Ishwar Singh

**Affiliations:** 1 Antimicrobial Drug Discovery and Development, The Robert Robinson Laboratories, Department of Chemistry, University of Liverpool, Liverpool L69 3BX, UK; r.malkawi@jadara.edu.jo (R.M.); james.weldon-bee@liverpool.ac.uk (J.W.-B.); edwin.kiptoo@liverpool.ac.uk (E.K.); sanjitpharma@gmail.com (S.D.); qian.zhang2@liverpool.ac.uk (Q.Z.); 2 Antimicrobial Pharmacodynamics and Therapeutics, Department of Pharmacology and Therapeutics, Institute of Systems, Molecular & Integrative Biology, University of Liverpool, William Henry Duncan Building, 6 West Derby St., Liverpool L7 8TX, UK; yinzhe.chen@liverpool.ac.uk; 3 School of Pharmacy, Jadara University, P.O. Box 733, Irbid 21110, Jordan; 4 Department of Chemistry and Materials Science, Xi’an-Jiaotong Liverpool University, Suzhou 215123, China; 5 School of Pharmacy, University of Lincoln, JBL Building, Beevor St., Lincoln LN6 7DL, UK; usariyer@gmail.com; 6 Singapore Eye Research Institute, Singapore 169857, Singapore; lakshminarayanan.rajamani@seri.com.sg; 7 Department of Pharmacy & Pharmaceutical Sciences, National University of Singapore, Singapore 117543, Singapore; 8 Ophthalmology and Visual Sciences Academic Clinical Program, Duke-NUS Graduate Medical School, Singapore 169857, Singapore

**Keywords:** teixobactin, macrolactamisation, cyclic peptides, antimicrobial peptides, macrocycle expansion, solid-phase peptide synthesis, methicillin-resistant *Staphylococcus aureus* (MRSA)

## Abstract

Teixobactin is a promising antibiotic that targets cell wall biosynthesis in Gram-positive bacteria and displays a low propensity for resistance; however, its structural complexity presents challenges for analogue development and optimisation. In this study, we investigated the effects of macrocycle size and replacement of the native depsipeptide linkage with an amide bond on antibacterial activity using a simplified Leu_10_-teixobactin scaffold. An amide-based macrocyclisation strategy was developed for efficient lactam formation using readily accessible amino acid building blocks, avoiding reliance on synthetically demanding modified diamino acids employed in other approaches. Two complementary synthetic routes provided access to a series of ten analogues, comprising linear and macrocyclised variants with systematic variation at position 8. Antibacterial activity was evaluated against methicillin-resistant *Staphylococcus aureus* (MRSA) and multidrug-resistant clinical isolates. While linear analogues exhibited weak or no measurable antibacterial activity, macrocyclised analogues retained measurable antibacterial activity, indicating that macrocyclisation is essential within this scaffold, whereas moderate expansion of the macrocycle was tolerated. The structure–activity relationships identified here demonstrate the suitability of a simplified Leu_10_-teixobactin framework and provide a platform for further optimisation of teixobactin-inspired antibiotics.

## 1. Introduction

The limited introduction of new antibiotics, together with the widespread use of existing agents, has contributed to the increasing prevalence of multidrug-resistant bacterial infections, which were associated with an estimated 4.71 million deaths globally in 2021 [1,2,3]. Although considerable efforts have been directed towards the discovery of new antibacterial agents, including antimicrobial peptides, bacterial resistance continues to emerge against multiple antibiotic classes, highlighting the urgent need for coordinated global action and new therapeutic strategies [4,5,6]. Among these, macrocyclic peptides continue to represent a diverse and expanding chemical space for antibiotic development [7]. As a result, there is sustained interest in identifying new antibacterial scaffolds with mechanisms of action that are less susceptible to resistance development. Lewis and his co-workers in 2015 reported a new naturally occurring antibiotic, teixobactin [8]. Teixobactin is highly potent against Gram-positive bacteria and thus far has not been associated with detectable resistance. Resistance to teixobactin is unlikely because it binds the highly conserved pyrophosphate moiety of the non-protein cell-wall precursors Lipid II and Lipid III, in contrast to vancomycin resistance which arises from the modification of the pentapeptide region of Lipid II; this multi-target, mutation-resistant mechanism underpins teixobactin’s superior activity and low resistance potential [9,10,11,12,13,14]. These properties make the teixobactin scaffold an attractive starting point for antibiotic development [15].

We have previously elucidated the mode of action of the teixobactin analogue _D_-Arg_4_-Leu_10_-teixobactin in bacterial membranes with Lipid II on a molecular level. The complex formed between _D_-Arg_4_-Leu_10_-teixobactin and labelled Lipid II was studied by solid-state NMR spectroscopy which revealed that _D_-Arg_4_-Leu_10_-teixobactin binds weakly with Lipid II in cellular membranes and is not solely responsible for potent antibacterial activity [16]. On the other hand, the analogue _D_-Arg_4_-Leu_10_-teixobactin forms micro clusters at a slow rate on the bacterial membrane surface. These observations, together with more recent visualisation and biophysical studies of teixobactin analogue assembly and membrane interactions, suggest that additional membrane-associated processes contribute to the antibacterial activity of teixobactin [16,17,18]. These findings indicate that the formation of membrane-associated clusters may sequester cell wall precursors and disrupt cell wall biosynthesis [16,19]. The synthesis of teixobactin is of moderate complexity, with the non-natural amino acid _L_-*allo*-End_10_ being the bottleneck in the synthesis. _L_-*allo*-End_10_ is not commercially available, laborious in synthesis and low-yielding upon incorporation into the peptide [20]. To address this limitation, several simplified synthetic strategies have been explored [21,22,23].

One effective approach to addressing this limitation has been substitution of _L_-*allo*-enduracididine at position 10 with leucine. Previous studies, including our own, demonstrated that the replacement of _L_-*allo*-enduracididine with leucine yields teixobactin analogues with potent antibacterial activity comparable to the natural product, indicating that a cationic residue at position 10 is not essential for activity [24]. Subsequent studies further confirmed the in vivo efficacy of _D_-Arg_4_-Leu_10_-teixobactin [25]. More recent work has also demonstrated potent in vivo antibacterial activity of teixobactin in an inhalation anthrax model [26].

Nowick’s group determined the X-ray crystal structure of truncated Arg_10_-teixobactin [27,28]. The crystal structure presented key interactions in the compound. The amide NH group of Ala_9_, Arg_10_, Ile_11_ and guanidinium side chain of Arg forms a cavity that binds to the chloride ion. Additionally, the -OH group of the Ser_7_ side chain forms a hydrogen bond with the NH of Ala_9_ [27]. The results hypothesised that the replacement of the lactone group of the macrocyclic ring by the lactam ring offers an extra amide NH, which strengthens the interaction with the chloride ion and eventually with the pyrophosphate group of Lipid II; however, they also found the presence of the β-methyl group present on the side chain of _D_-Threonine (_D_-Thr) at position 8 to be crucial in maintaining antibacterial activity [28,29].

The substitution of _D_-Thr8 with _D_-2,3-diaminopropionic acid (_D_-Dap) derivatives bearing a β-methyl substituent was used to probe the role of ring size and linkage chemistry in Arg_10_ teixobactin analogues. These analogues replaced the native lactone with a lactam while retaining comparable activity to Arg_10_-teixobactin, demonstrating that ester–amide isosteric substitution is tolerated. Building on this concept, the authors synthesised seven ring-expanded Arg_10_-teixobactin analogues incorporating β^3^-homo amino acids, proposing that increased ring size may better accommodate the pyrophosphate moiety of Lipid II. Importantly, the retention of the β-methyl substituent on the Dab/Dap residue was essential for maintaining antibacterial activity [29].

Previous work by Scioli et al. using Arg_10_-teixobactin analogues showed that replacing the native depsipeptide linkage with a total lactam ring maintained antibacterial activity and, based on structural considerations, was proposed to enhance interactions with Lipid II through additional amide functionality [30]. These results encourage further investigation of side-chain modifications and alternative macrocyclisation approaches to enhance the physicochemical and biological characteristics of teixobactin analogues.

Despite these advances, the combined effects of macrocycle size expansion and replacement of the native depsipeptide linkage with an amide bond within a simplified Leu_10_-teixobactin scaffold have not been systematically investigated.

**Figure 1:**
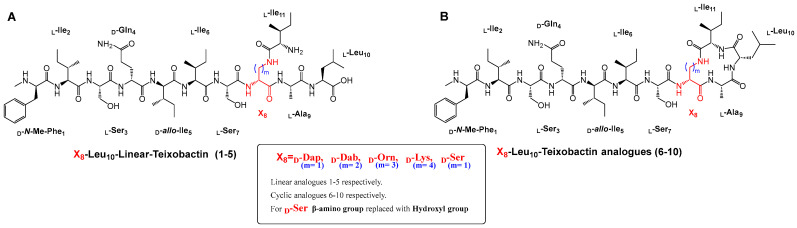
(**A**) Linear Leu_10_-teixobactin analogues (**1**–**5**) incorporating _D_-amino acid substitutions at position 8 (X_8_). (**B**) Corresponding cyclised Leu_10_-teixobactin analogues (**6**–**10**) featuring ring closure through the position-8 residue. Substituents at X_8_ include _D_-Dap, _D_-Dab, _D_-Orn, _D_-Lys, and _D_-Ser, with varying side-chain lengths (highlighted in red).

We investigated the effect of ring expansion of teixobactin on antibacterial activity (Figure 1) based on Leu_10_-teixobactin. We chose Leu_10_-teixobactin as the core template due to its superior antibacterial activity (eight-fold) in comparison to Arg_10_-teixobactin against MRSA [15,24]. Herein, we report the synthesis and evaluation of a series of ring-expanded teixobactin analogues based on our Leu_10_-teixobactin scaffold, enabling systematic assessment of macrocycle size and linkage type on antibacterial activity (Figure 1A,B).

## 2. Materials and Methods

### 2.1. General Reagents, Analytical and Instrumentation

All amino acids, 1-[Bis(dimethylamino)methylene]-1H-1,2,3 triazolo[4,5-b]pyridinium3-oxidhexafluorophosphate (HATU), Phenylsilane (PhSiH_3_), Diisopropylethylamine (DIPEA), Tritylchloride 4-(Dimethylamino)pyridine(DMAP), Tetrakis(triphenylphosphine)palladium(0) Pd(PPh_3_)_4_, Ethyl cyano(hydroxyimino)acetate (Oxyma Pure), Diisopropylcarbodiimide (DIC) and Triisopropylsilane (TIS), were purchased from Fluorochem, Hadfield, UK. Dimethylformamide (DMF), peptide synthesis grade, was purchased from Rathburn chemicals, Walkerburn, UK. Triethylamine, Diethyl ether (Et_2_O), Dimethylsulfoxide (DMSO), Dichloromethane (DCM), formic acid 98–100% purity, water (HPLC grade) and Acetonitrile (HPLC grade) were purchased from Fisher Scientific, Loughborough, UK. 2-chlorotrityl chloride resin (manufacturer’s loading: 1.60 mmol Cl^−^/g) was purchased from Iris Biotech GmbH, Marktredwitz, Germany. All chemicals were used without further purification. Compounds were analysed on a Thermo Scientific Dionex Ultimate 3000 RP-HPLC (Thermo Scientific, Hemel Hempstead, UK) equipped with a Phenomenex Gemini NX C18 110 Å (150 × 4.6 mm) column (Phenomenex, Macclesfield, UK) using the following buffer systems: A: 0.1% HCOOH in water; B: ACN using a flow rate of 1 mL/min. The column was flushed with 95% A for 5 min prior to an injection and was flushed for 5 min with 95% B and 5% A after the run was finished. HRMS spectra were recorded on two machines: a Thermo Scientific Q Exactive Plus Orbitrap Mass Spectrometer and an Agilent QTOF 7200 Mass Spectrometer (Agilent, Santa Clara, CA, USA) in the positive ion mode. NMR was carried out at 27 °C on a Bruker Avance III HD 500 MHz spectrometer (Bruker, Billerica, MA, USA) equipped with a room-temperature broadband probe.

### 2.2. Synthesis of Amino Acid Dimers (***ii***, ***iv***, ***vi***, ***viii***)

Boc-Ile-OH (1.0 eq.) and the corresponding Fmoc-protected _D_-amino acids (Fmoc-_D_-Dap-OH (**i**), Fmoc-_D_-Dab-OH (**iii**), Fmoc-_D_-Orn-OH (**v**), or Fmoc-_D_-Lys-OH (**vii**)) were dissolved in DMF (2 mL). HATU (0.8 eq.) and DIPEA (3.0 eq.) were added, and the reaction mixture was stirred for 1 h at room temperature. The reaction was quenched with 1 M citric acid in water, and the products were extracted with dichloromethane and purified by normal-phase chromatography (see Scheme 1 and Supplementary Scheme S1, Table S1, Figures S1–S16).

### 2.3. Synthesis of Fmoc-Alloc Protected Amino Acid Building Blocks (***ix–xii***)

Fmoc-protected amino acids (Fmoc-D-Dap-OH (**i**), Fmoc-D-Dab-OH (**iii**), Fmoc-D-Orn-OH (**v**), or Fmoc-D-Lys-OH (**vii**); 1.0 eq.) were dissolved in THF (10 mL), followed by the addition of NaHCO_3_ (5.0 eq.). Allyl chloroformate (1.5 eq.) was added dropwise, and the reaction mixture was stirred at room temperature overnight. Reaction progress was monitored by TLC. Upon completion, the reaction mixture was diluted with water (50 mL) and acidified to pH 2 using 1 M HCl. The aqueous phase was extracted with DCM (3 × 50 mL), and the combined organic layers were dried over anhydrous Na_2_SO_4_, filtered, and concentrated under reduced pressure. The crude products were purified by silica gel column chromatography using DCM/MeOH as the eluent to afford the corresponding Fmoc- and Alloc-protected amino acid building blocks. Product identity was confirmed by LC–MS, and the compounds were used directly for subsequent peptide synthesis (see Scheme 2 and Supplementary Scheme S2, Table S2, Figures S17–S20).

### 2.4. Synthesis of Teixobactin Analogues Using a Dimer Strategy

Teixobactin analogues were synthesised on 2-chlorotrityl chloride resin using standard Fmoc solid-phase peptide synthesis protocols. Fmoc-Leu-OH (4 eq.) was coupled using DIPEA (8 eq.) in DCM, followed by Fmoc deprotection with 20% piperidine in DMF. Subsequent amino acids were coupled using Fmoc-protected amino acids and DIC/Oxyma activation under microwave irradiation. Fmoc deprotection was performed using 20% piperidine in DMF. The dimer building block was coupled at position 8 under standard coupling conditions. Global deprotection and resin cleavage were performed using TFA/TIS/H_2_O (95:2.5:2.5, *v*/*v*/*v*). Macrocyclisation was carried out in solution using HATU (3 eq.) and DIPEA (10 eq.) in DMF for 1 h (See Scheme 3 and Supplementary Scheme S3).

### 2.5. Synthesis of Teixobactin Analogues Using Alloc-Protected Strategy

Teixobactin analogues incorporating Alloc-protected amino acids were synthesised on 2-chlorotrityl chloride resin using standard Fmoc solid-phase peptide synthesis protocols. Following assembly of the linear peptide sequence, selective removal of the Alloc protecting group was carried out using Pd(PPh_3_)_4_ and phenylsilane in dichloromethane. Subsequent amino acid coupling, macrolactamisation and global deprotection were performed using standard solid-phase peptide synthesis procedures. All final peptide analogues were purified by RP-HPLC prior to biological evaluation and were obtained with analytical purities ranging between 90 and 98% as determined by HPLC analysis (see Scheme 4, Supplementary Scheme S4, Table S3, Figures S21–S40).

### 2.6. Antimicrobial Susceptibility Testing

Bacterial cultures were grown overnight in Mueller–Hinton Agar (MHA) plates and adjusted to a final concentration of 10^5^–10^6^ CFU/mL. An amount of 100 µL of inoculum in Mueller–Hinton broth (MHB) was mixed with an equal volume of peptides (dissolved in MHB) at 2× their concentration in a 96-well plate. In parallel experiments, MIC values were determined in media containing polysorbate 80 (0.002%, *v*/*v*) to prevent non-specific adsorption of the peptides to plastic surfaces. The final peptide concentrations ranged from 0.0625 to 32 µg/mL. Positive and negative controls contained 200 µL of broth with/without inoculum without peptide dissolved, respectively. The 96-well plates were incubated at 37 °C for 24 h. All experiments were performed in triplicate, and the MIC was determined as the lowest concentration at which no visible growth was observed.

## 3. Results

### 3.1. Establishing Synthetic Routes for Ring-Expanded Teixobactin Analogues

**Figure 2:**
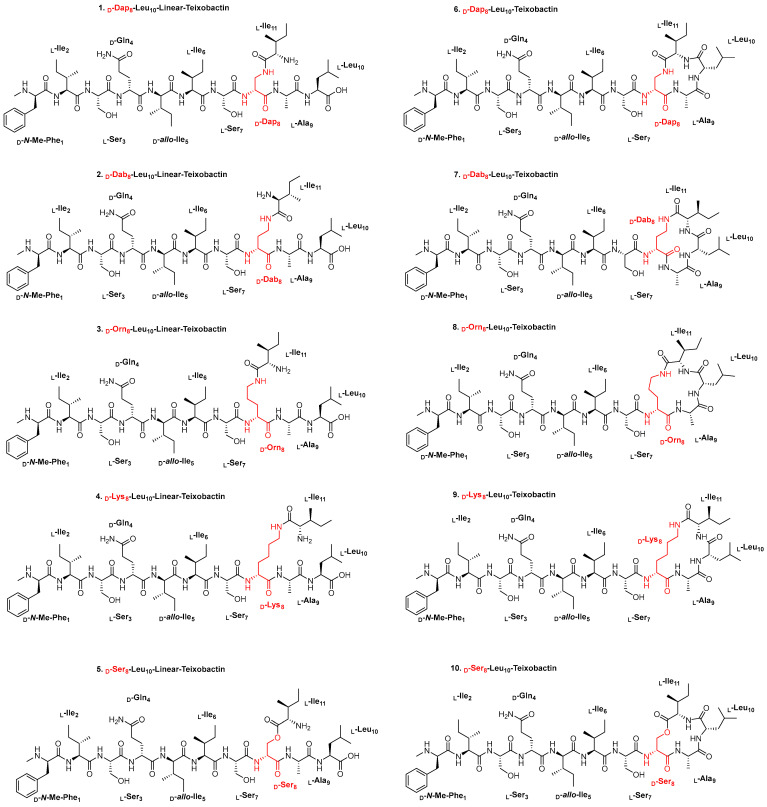
Uncyclised and cyclised analogues of Leu_10_-teixobactin with varying side-chain lengths at position 8 (shown in red).

A series of teixobactin analogues based on the Leu_10_-teixobactin scaffold were designed to enable systematic evaluation of macrocycle ring size and linkage type (Figure 2). Ring expansion was achieved by substitution of _D_-threonine at position 8 with amino acids bearing primary amine side chains of increasing length, enabling macrolactam formation with Ile_11_.

**Scheme 1:**
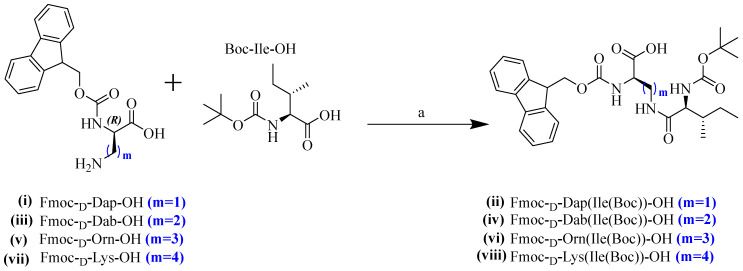
Synthesis of Fmoc-protected amino acid dimer building blocks via coupling of Boc-Ile-OH to Fmoc-_D_-amino acids with varying side-chain lengths (**m = 1–4**). Reagents and conditions: (a) HATU (0.8 equiv.), DIPEA (3 equiv.), DMF, 1 h.

An initial synthetic approach employed pre-formed amino acid dimer building blocks to introduce flexibility at the cyclisation site. Specifically, Fmoc-protected dimer building blocks incorporating _D_-Dap, _D_-Dab, _D_-Orn, or _D_-Lys at position 8 were synthesised separately (**ii**, **iv**, **vi**, **viii**) (Scheme 1) and subsequently incorporated during solid-phase peptide synthesis (Scheme 3). The identity of the dimer building blocks was confirmed by NMR spectroscopy and high-resolution mass spectrometry (see Supplementary Schemes S1 and S2, Section SIII).

The synthesised dimers were subsequently incorporated during solid-phase peptide synthesis on 2-chlorotrityl chloride resin, followed by global deprotection and solution-phase macrolactamisation to afford the corresponding macrocyclised analogues.

Although incorporation of the dimer building blocks proceeded as expected, selective removal of the Boc protecting group required for macrolactam formation was inefficient at a late stage of the synthesis, necessitating full global deprotection prior to cyclisation. Global deprotection at this stage removes all acid-labile-protecting groups, increasing the number of reactive functionalities present during macrolactam formation and thereby raising the risk of competing side reactions. As a result, the dimer-based strategy afforded the desired macrocyclised products in low isolated yields (8–12%).

To overcome these limitations, an alternative strategy employing Alloc-protected amino acid building blocks was developed. The Alloc protecting group enables orthogonal and quantitative side-chain deprotection, allowing selective liberation of the amine required for cyclisation while all other functionalities remain protected.

**Scheme 2:**
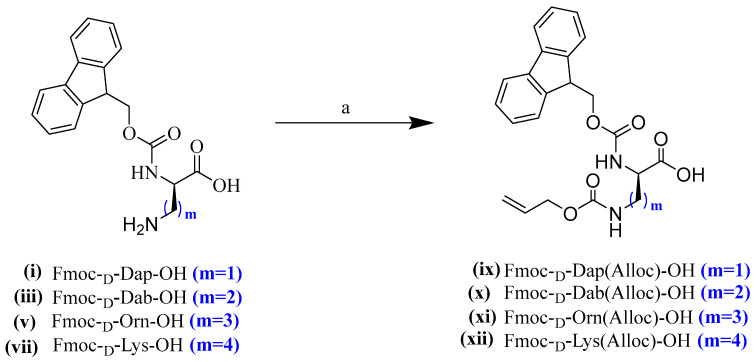
Synthesis of Fmoc-Alloc building blocks. (a) 5 eq. NaHCO_3_, 1.5 eq. Allyl chloroformate, THF, 18 h. **m = 1–4**, indicates increase in chain length of different residues used.

**Scheme 3:**
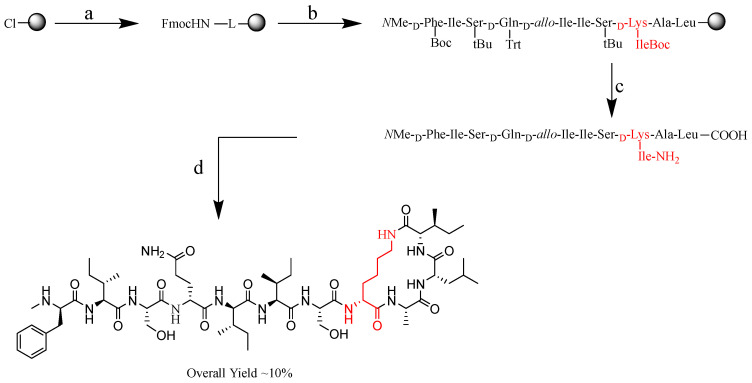
Total synthesis of analogue **9** using a dimer-based strategy starting from 2-chlorotrityl chloride resin: (a) 4 eq. of Fmoc-Leu-OH/8 eq. DIPEA, in DCM, 4 h, 20% piperidine in DMF. (b) Fmoc/Trt/Boc-AA(PG)-OH (AA = amino acid, PG = protecting group), DIC/Oxyma in microwave, followed by 20% piperidine in DMF to remove the Fmoc; position 8 coupled with the dimer D-Fmoc-Lys-Ile-Boc. (c) Full deprotection was conducted using TFA:TIS:H_2_O, 95:2.5:2.5%. (d) Macrocyclisation was performed using 3 eq. HATU and 10 eq. DIPEA in DMF for 1 h.

Accordingly, the Alloc-protected amino acid building blocks Fmoc-_D_-Dap(Alloc)-OH (**ix**), Fmoc-_D_-Dab(Alloc)-OH (**x**), Fmoc-_D_-Orn(Alloc)-OH (**xi**), and Fmoc-_D_-Lys(Alloc)-OH (**xii**) were synthesised in-house to enable orthogonal side-chain protection (Scheme 2).

**Scheme 4:**
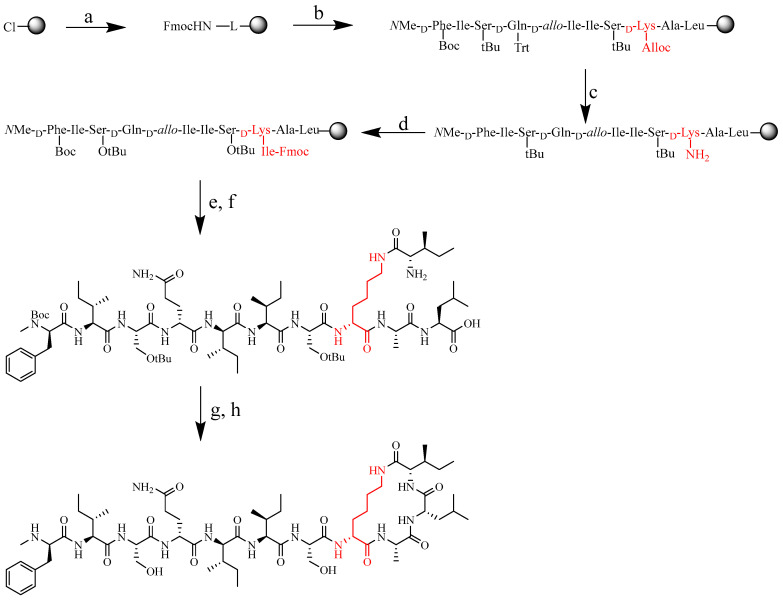
Total synthesis of analogue **9** via Alloc strategy starting from 2-chlorotrityl chloride resin: (a) 4 eq. of Fmoc-Leu-OH/8 eq. DIPEA, in DCM, 4 h; (b) 20% piperidine in DMF 2 × 10 min. Full synthesis was performed using an automated synthesiser; 4 eq. Fmoc/Boc-AA(PG)-OH (AA = amino acid, PG = protecting group), 4 eq. HATU, 8 eq. DIPEA, RT for 1 h, followed by 20% piperidine in DMF to remove the Fmoc. (c) Selective deprotection of Alloc was performed using Pd(PPh_3_)_4_ (0.2 eq.) + 24 eq. PhSiH_3_ in DCM, 2 × 30 min; (d) 4 eq. of Fmoc-Ile-OH/4 eq. HATU/8 eq. DIPEA in DMF, 1 h; (e,f) 20% piperidine in DMF 2 × 10 min, followed by partial cleave with TFA:TIS:DCM = 2:2:96, 1 h; (g,h) 1 eq. HATU/10 eq. DIPEA in DMF, 1 h, followed by full cleave with TFA:TIS:H_2_O = 95:2.5:2.5, 1 h (See Supplementary Scheme S4).

In this strategy, the Alloc-protected amino acid was incorporated into the growing peptide chain by standard Fmoc solid-phase peptide synthesis. Selective removal of the Alloc protecting group was then performed on resin to liberate the side-chain amine required for macrolactam formation, after which the subsequent amino acid residue (Ile) was coupled using standard SPPS conditions. This orthogonal deprotection sequence enabled partial cleavage from the resin prior to macrolactamisation, ensuring that cyclisation occurred in the presence of a single reactive amine and thereby favouring clean ring closure (Scheme 4).

Compared to the dimer-based approach, the Alloc-protected strategy enabled controlled macrolactam formation under standard amide coupling conditions (Scheme 4). This resulted in improved overall efficiency and reproducibility, affording the macrocyclised analogues in higher isolated yields (16–18%). Using these synthetic strategies, a total of ten teixobactin analogues were prepared comprising five linear analogues (**1**–**5**) and five macrocyclised analogues (**6**–**10**).

### 3.2. Design and Preparation of Linear and Macrocyclised Teixobactin Analogues

This analogue set enabled direct comparison of linear and macrocyclic peptides, allowing systematic evaluation of macrocycle expansion and replacement of the native depsipeptide linkage with an amide bond on antibacterial activity. In total, ten Leu_10_-teixobactin analogues were prepared, comprising five linear peptides (**1**–**5**) and five corresponding macrocyclic analogues (**6**–**10**) (Figure 2).

Variation at position 8 was achieved by substitution of _D_-threonine with commercially available amino acids bearing primary amine side chains of increasing length, including _D_-2,3-diaminopropionic acid (_D_-Dap), _D_-2,4-diaminobutyric acid (_D_-Dab), D-ornithine (_D_-Orn), and _D_-lysine (_D_-Lys). These substitutions enabled controlled expansion of the macrocycle from the native 13-membered ring to larger macrolactams in the cyclised analogues. A _D_-serine-containing analogue was included as a control to assess the influence of β-methyl substitution at position 8. For this analogue, a _D_-serine residue was incorporated at position 8, the linear peptide chain was assembled by standard SPPS, and esterification with Ile was performed to afford the corresponding depsipeptide (**10**).

Linear analogues were synthesised without cyclisation to assess the contribution of the macrocycle to antibacterial activity, while the corresponding macrocyclised analogues incorporated an amide linkage between the side chain at position 8 and the carboxylate of Ile_11_. This design enabled direct comparison of linear versus macrocyclised peptides and systematic assessment of the impact of macrocycle expansion on antibacterial activity.

### 3.3. Antimicrobial Activity of Teixobactin Analogues

The antibacterial activity of the synthesised teixobactin analogues was initially evaluated against methicillin-resistant *Staphylococcus aureus* (MRSA) ATCC 33591 to establish baseline potency and assess the impact of macrocyclisation and ring size. The minimum inhibitory concentration (MIC) values for the linear analogues (**1**–**5**) and macrocyclised analogues (**6**–**10**) are summarised in Table 1.

The linear analogues exhibited weak or no antibacterial activity against MRSA ATCC 33591, with MIC values ≥ 32 μg/mL. In contrast, macrocyclised analogues displayed measurable antibacterial activity, with _D_-Dap_8_-Leu_10_-teixobactin (**6**), _D_-Dab_8_-Leu_10_-teixobactin (**7**), and _D_-Lys_8_-Leu_10_-teixobactin (**9**) exhibiting MIC values of 4 μg/mL. Within the macrocyclised series, variation in the amino acid at position 8 was generally tolerated, with the majority of the analogues displaying comparable antibacterial activity. _D_-Dap_8_-Leu_10_-teixobactin (**6**), _D_-Dab_8_-Leu_10_-teixobactin (**7**), _D_-Orn_8_-Leu_10_-teixobactin (**8**) and _D_-Lys_8_-Leu_10_-teixobactin (**9**) exhibited similar antibacterial activity across the tested strains. Notably, _D_-Ser_8_-Leu_10_-teixobactin (**10**), which retains a comparable ring size to the _D_-Dap analogue but incorporates a depsipeptide linkage, also exhibited reduced activity.

These data indicate that macrocyclisation is required to retain antibacterial activity in this analogue series and that increased macrocycle size is also tolerated.

Based on the activity observed against MRSA ATCC 33591, selected macrocyclised analogues (**6**–**10**) were further evaluated against a panel of multidrug-resistant (MDR) *Staphylococcus aureus* clinical isolates to assess whether antibacterial activity was retained across strains with distinct susceptibility profiles. The antibacterial activity of these analogues, alongside clinically used antibiotics, is summarised in Table 2.

Across the MDR clinical isolates tested, the macrocyclised teixobactin analogues exhibited variable antibacterial activity, with MIC values differing between strains. Analogues **6**, **7**, and **9** generally displayed lower MIC values across multiple isolates relative to the remaining macrocyclised analogues. In contrast, _D_-Orn_8_-Leu_10_-teixobactin (**8**) showed reduced potency across several isolates, consistent with increased macrocycle size. The reduced activity of _D_-Ser_8_-Leu_10_-teixobactin (**10**) likely reflects both the loss of the β-methyl substituent and the change in hydrogen-bonding geometry introduced by the depsipeptide linkage. Notably, several clinical isolates displayed reduced susceptibility to linezolid, cefotaxime, levofloxacin, and ampicillin, whereas the macrocyclised teixobactin analogues retained measurable antibacterial activity across the tested MDR strains.

Collectively, these results show that macrocyclisation is required for antibacterial activity in this analogue series and that increased macrocycle size is tolerated with respect to our Leu_10_-teixobactin scaffold.

## 4. Discussion

In this study, we evaluated how macrocycle size and the replacement of the native depsipeptide linkage with an amide bond influence antibacterial activity within a simplified Leu_10_-teixobactin scaffold. Two complementary synthetic strategies enabled the preparation of both linear and macrocyclised analogues, allowing direct comparison of macrocyclisation, ring expansion, and linkage type within a consistent structural framework.

The loss of antibacterial activity in the linear analogues highlights the critical role of macrocyclisation in maintaining antibacterial function in teixobactin-derived scaffolds. Consistent with prior studies, the removal of the macrocycle resulted in a marked reduction in activity, underscoring the importance of conformational constraint for activity within this scaffold.

Native teixobactin contains the non-proteinogenic amino acid _L_-*allo*-enduracididine at position 10, which has been proposed to contribute to antibacterial activity through target interactions. However, our previous work demonstrated that substitution of _L_-*allo*-enduracididine with leucine does not abolish antibacterial activity, enabling the development of a simplified and synthetically accessible Leu_10_-teixobactin framework. Building on this finding, the present study explores whether macrocyclisation within this Leu_10_-teixobactin scaffold remains compatible with antibacterial activity and enables evaluation of macrocycle size–activity relationships.

Systematic expansion of the macrocycle through variation in the amino acid at position 8 revealed a relationship between macrocycle size and antibacterial activity. Analogues incorporating shorter side-chain linkers, such as _D_-Dap and _D_-Dab, retained antibacterial potency, indicating that moderate macrocycle expansion is tolerated. Further expansion using _D_-Orn and _D_-Lys resulted in modest variations in antibacterial activity, with MIC values remaining within a comparable range (4–8 µg/mL) across the series, suggesting that increased macrocycle size is broadly tolerated in this analogue set.

Notably, _D_-Ser_8_-Leu_10_-teixobactin (analogue **10**) was included as a depsipeptide-linked comparator within the simplified Leu_10_-teixobactin scaffold and provides insight into the importance of linkage type independent of macrocycle size. Unlike the amide-linked analogues, analogue **10** incorporates a depsipeptide linkage analogous to native teixobactin but lacks the β-methyl substituent present in _D_-threonine. Despite retaining a macrocycle size comparable to the _D_-Dap analogue, this compound exhibited reduced antibacterial activity, indicating that both the nature of the linkage and side-chain substitution at position 8 influence activity. The absence of the β-methyl group in serine may influence local conformational preferences or hydrogen-bonding interactions, contributing to the observed reduction in activity.

Previous studies have reported macrocyclisation strategies employing methylated diamino acids to facilitate lactam formation, often in combination with alternative residues at position 10. While effective, these approaches rely on synthetically demanding building blocks. In contrast, the present work demonstrates that lactamisation can be achieved within a simplified Leu_10_-teixobactin scaffold using readily accessible amino acids, while retaining measurable antibacterial activity. This highlights the potential of more synthetically tractable macrocyclisation strategies for the development of teixobactin-inspired antibiotics.

Evaluation against multidrug-resistant clinical *Staphylococcus aureus* isolates revealed that selected macrocyclised analogues retained comparable antibacterial activity to that of the reference MRSA strain. While diminished activity was observed for some clinical antibiotics across these isolates, the teixobactin analogues maintained measurable antibacterial effects, consistent with a mode of action distinct from those of clinical antibiotics. Together, these findings demonstrate that controlled macrocyclisation within a simplified teixobactin scaffold can retain antibacterial activity and provide insight into the structural features governing potency.

## 5. Conclusions

In summary, we report the synthesis of ring-expanded teixobactin analogues using an amide-based macrocyclisation strategy within a simplified Leu_10_-teixobactin scaffold. This approach enables controlled macrolactam formation using readily accessible amino acid building blocks and avoids reliance on synthetically complex methylated diamino acids employed in other lactamisation strategies.

The testing of linear and macrocyclised analogues established that macrocyclisation is essential for antibacterial activity, with moderate expansion of the macrocycle being tolerated. A comparison of amide-linked and depsipeptide-linked analogues highlighted the influence of both macrocycle geometry and side-chain substitution at position 8 on activity. The methodology and structure–activity relationships reported here provide a foundation for future SAR exploration and the rational design of teixobactin-inspired antibiotics. Future studies will focus on further optimisation and evaluation of the toxicity and pharmacological properties of these analogues.

## Figures and Tables

**Table 1:** Antibacterial activity of teixobactin analogues against methicillin-resistant *Staphylococcus aureus* (MRSA) ATCC 33591. MIC: minimum inhibitory concentration (µg/mL). All MIC values were determined in triplicate.

ANALOGUE NO.	NAME/COMPOSITION	MIC AGAINST MRSA ATCC 33591	MACROCYCLE SIZE
**1**	_D_-Dap_8_-Leu_10_-Linear Teixobactin	32	-
**2**	_D_-Dab_8_-Leu_10_-Linear Teixobactin	>32	-
**3**	_D_-Orn_8_-Leu_10_-Linear Teixobactin	>32	-
**4**	_D_-Lys_8_-Leu_10_-Linear Teixobactin	>32	-
**5**	_D_-Ser_8_-Leu_10_-Linear Teixobactin	>32	-
**6**	_D_-Dap_8_-Leu_10_-Teixobactin	4	13
**7**	_D_-Dab_8_-Leu_10_-Teixobactin	4	14
**8**	_D_-Orn_8_-Leu_10_-Teixobactin	8	15
**9**	_D_-Lys_8_-Leu_10_-Teixobactin	4	16
**10**	_D_-Ser_8_-Leu_10_-Teixobactin	8	13

**Table 2:** Antibacterial activity of teixobactin analogues and clinically used antibiotics against MDR *Staphylococcus aureus* clinical isolates. The colours represent the MIC activity profile: 1–2 µg/mL (potent activity, green), 4–8 µg/mL (moderate activity, yellow), and ≥16 µg/mL (poor activity, red).

	*Staphylococcus aureus*			
	NR 45914	NR 45926	NR 45930	NR 46062
Analogue **6**	8	4	2	2
Analogue **7**	4	4	2	2
Analogue **8**	8	8	4	2
Analogue **9**	4	8	4	2
Analogue **10**	8	8	4	4
Vancomycin	1	0.5	1	2
Linezolid	2	32	4	16
Cefotaxime	4	16	8	16
Levofloxacin	1	4	>32	32
Ampicillin	2	32	8	8
Daptomycin	2	4	2	2

## Data Availability

The data presented in this study are available in the Supplementary Materials of this article.

## Supplementary Materials

The following supporting information can be downloaded at: https://www.mdpi.com/article/10.3390/biom16070970/s1. I: Materials; II: Equipment Used for the Analysis and Purification of Compounds; III: Synthesis & Analysis of Dimeric Amino Acid Building Blocks (HRMS, 1H NMR, 13C NMR)2. (Scheme S1, Figures S1–S16, Table S1); IV: Synthesis of Alloc-Protected Amino Acid Building Blocks. (Scheme S2, Figures S17–S20, Table S2); V: Total Synthesis of Teixobactin Analogues via Dimer Approach. (Scheme S3); VI: Total Synthesis of Teixobactin Analogues via an Alloc-Protected Strategy. (Scheme S4, Table S3); VII: HPLC/Mass Analysis for the Molecules 1–10. (Figures S21–S40); VIII: MIC Testing (Screening).

## References

[1] Butler M.S., Gigante V., Sati H., Paulin S., Al-Sulaiman L., Rex J.H., Fernandes P., Arias C.A., Paul M., Thwaites G.E. (2022). Analysis of the Clinical Pipeline of Treatments for Drug-Resistant Bacterial Infections: Despite Progress, More Action Is Needed. Antimicrob. Agents Chemother..

[2] Ntallis C., Martin N.I., Edwards A.M., Weingarth M. (2025). Bacterial Cell Envelope-Targeting Antibiotics. Nat. Rev. Microbiol..

[3] Naghavi M., Vollset S.E., Ikuta K.S., Swetschinski L.R., Gray A.P., Wool E.E., Robles Aguilar G., Mestrovic T., Smith G., Han C. (2024). Global Burden of Bacterial Antimicrobial Resistance 1990–2021: A Systematic Analysis with Forecasts to 2050. Lancet.

[4] WHO Antimicrobial Resistance Division (AMR) (2025). Global Antibiotic Resistance Surveillance Report 2025.

[5] Zucca M., Savoia D. (2010). The Post-Antibiotic Era: Promising Developments in the Therapy of Infectious Diseases. Int. J. Biomed. Sci..

[6] The Lancet (2024). Antimicrobial Resistance: An Agenda for All. Lancet.

[7] Garcia Jimenez D., Poongavanam V., Kihlberg J. (2023). Macrocycles in Drug Discovery—Learning from the Past for the Future. J. Med. Chem..

[8] Ling L.L., Schneider T., Peoples A.J., Spoering A.L., Engels I., Conlon B.P., Mueller A., Schäberle T.F., Hughes D.E., Epstein S. (2015). A New Antibiotic Kills Pathogens without Detectable Resistance. Nature.

[9] Piddock L.J.V. (2015). Teixobactin, the First of a New Class of Antibiotics Discovered by IChip Technology?. J. Antimicrob. Chemother..

[10] Homma T., Nuxoll A., Gandt A.B., Ebner P., Engels I., Schneider T., Götz F., Lewis K., Conlon B.P. (2016). Dual Targeting of Cell Wall Precursors by Teixobactin Leads to Cell Lysis. Antimicrob. Agents Chemother..

[11] von Nussbaum F., Süssmuth R.D. (2015). Multiple Attack on Bacteria by the New Antibiotic Teixobactin. Angew. Chem. Int. Ed..

[12] Puls J.S., Brajtenbach D., Schneider T., Kubitscheck U., Grein F. (2023). Inhibition of Peptidoglycan Synthesis Is Sufficient for Total Arrest of Staphylococcal Cell Division. Sci. Adv..

[13] Lewis K., Lee R.E., Brötz-Oesterhelt H., Hiller S., Rodnina M.V., Schneider T., Weingarth M., Wohlgemuth I. (2024). Sophisticated Natural Products as Antibiotics. Nature.

[14] Liu Y., Liu Y., Chan-Park M.B., Mu Y. (2017). Binding Modes of Teixobactin to Lipid II: Molecular Dynamics Study. Sci. Rep..

[15] Guo C., Mandalapu D., Ji X., Gao J., Zhang Q. (2018). Chemistry and Biology of Teixobactin. Chem. Eur. J..

[16] Shukla R., Medeiros-Silva J., Parmar A., Vermeulen B.J.A., Das S., Paioni A.L., Jekhmane S., Lorent J., Bonvin A.M.J.J., Baldus M. (2020). Mode of Action of Teixobactins in Cellular Membranes. Nat. Commun..

[17] Morris M.A., Vallmitjana A., Grein F., Schneider T., Arts M., Jones C.R., Nguyen B.T., Hashemian M.H., Malek M., Gratton E. (2022). Visualizing the Mode of Action and Supramolecular Assembly of Teixobactin Analogues in Bacillus Subtilis. Chem. Sci..

[18] Herron N., Vanegas J.M., Jeffy J., Srinivasan S., Kasson P., Werb J., Zeno W.F., Barreto Gomes D.E., Chen S.-C., Isralewitz B. (2026). BPS2026—Decoding Teixobactin-Lipid II Binding: How Local Environments and Hydration Shape Interaction Strength. Biophys. J..

[19] Shukla R., Lavore F., Maity S., Derks M.G.N., Jones C.R., Vermeulen B.J.A., Melcrová A., Morris M.A., Becker L.M., Wang X. (2022). Teixobactin Kills Bacteria by a Two-Pronged Attack on the Cell Envelope. Nature.

[20] Craig W., Chen J., Richardson D., Thorpe R., Yuan Y. (2015). A Highly Stereoselective and Scalable Synthesis of L-Allo-Enduracididine. Org. Lett..

[21] Gunjal V.B., Thakare R., Chopra S., Reddy D.S. (2020). Teixobactin: A Paving Stone toward a New Class of Antibiotics?. J. Med. Chem..

[22] Kumari D., Nagendra G. (2025). Synthetic Strategies and Biological Activities of Teixobactin and Its Analogs: A Review. Curr. Top. Med. Chem..

[23] Jin K., Po K.H.L., Wang S., Reuven J.A., Wai C.N., Lau H.T., Chan T.H., Chen S., Li X. (2017). Synthesis and Structure-Activity Relationship of Teixobactin Analogues via Convergent Ser Ligation. Bioorg. Med. Chem..

[24] Parmar A., Iyer A., Prior S.H., Lloyd D.G., Leng Goh E.T., Vincent C.S., Palmai-Pallag T., Bachrati C.Z., Breukink E., Madder A. (2017). Teixobactin Analogues Reveal Enduracididine to Be Non-Essential for Highly Potent Antibacterial Activity and Lipid II Binding. Chem. Sci..

[25] Parmar A., Lakshminarayanan R., Iyer A., Mayandi V., Leng Goh E.T., Lloyd D.G., Chalasani M.L.S., Verma N.K., Prior S.H., Beuerman R.W. (2018). Design and Syntheses of Highly Potent Teixobactin Analogues against *Staphylococcus aureus*, Methicillin-Resistant *Staphylococcus aureus* (MRSA), and Vancomycin-Resistant Enterococci (VRE) In Vitro and In Vivo. J. Med. Chem..

[26] Lawrence W.S., Peel J.E., de Winter R., Ling L.L., Nitti A.G., Peoples A.J., Shukla R., MacGillavry H.D., Heine H.S., Hensel M.E. (2025). Teixobactin: A Resistance-Evading Antibiotic for Treating Anthrax. ACS Infect. Dis..

[27] Yang H., Wierzbicki M., Du Bois D.R., Nowick J.S. (2018). X-Ray Crystallographic Structure of a Teixobactin Derivative Reveals Amyloid-like Assembly. J. Am. Chem. Soc..

[28] Yang H., Du Bois D.R., Ziller J.W., Nowick J.S. (2017). X-Ray Crystallographic Structure of a Teixobactin Analogue Reveals Key Interactions of the Teixobactin Pharmacophore. Chem. Commun..

[29] Yang H., Pishenko A.V., Li X., Nowick J.S. (2020). Design, Synthesis, and Study of Lactam and Ring-Expanded Analogues of Teixobactin. J. Org. Chem..

[30] Scioli G., Marinaccio L., Bauer M., Kamysz W., Parmar A., Newire E., Singh I., Stefanucci A., Mollica A. (2023). New Teixobactin Analogues with a Total Lactam Ring. ACS Med. Chem. Lett..

