# Supplementary material for: Understanding the Role of Macrocycle Size and Amide Linkage in Teixobactin Analogues
Source: Biomolecules. 2026 Jul 1;16(7):970. doi: 10.3390/biom16070970 (PMC13406156; doi:10.3390/biom16070970)
Supplement: Supplementary file 1 [file biomolecules-16-00970-s001.zip › biomolecules-4282711-supplementary.pdf]

Understanding the Role of Macrocycle Size and Amide Linkage in Teixobactin Analogues

Ruba Malkawi[1,2,3], James Weldon-Bee[1,2], Edwin Kiptoo[1,2], Sanjit Das[1,2] Yinzhe Chen[2,4], Abhishek Iyer[5], Rajamani Lakshminarayanan[6], Qian Zhang[1,4], Anish Parmar[1,2,*] and Ishwar Singh[1,2,*]

1 Department of Chemistry, University of Liverpool, Liverpool L69 7ZD, U.K.
2 Antimicrobial Pharmacodynamics and Therapeutics, Department of Pharmacology and Therapeutics, Institute of Systems, Molecular and Integrative Biology, University of Liverpool, Liverpool L69 7BE, U.K. William Henry Duncan Building, University of Liverpool, Liverpool L7 8TX, U.K.
3 Present address: School of Pharmacy, Jadara University, P.O. Box 733, Irbid 21110, Jordan
4 Department of Chemistry and Materials Science, Xi'an-Jiaotong Liverpool University, Suzhou, Jiangsu Province, 215123, PR China.
5 School of Pharmacy, JBL Building, University of Lincoln, Beevor St. Lincoln, LN67DL, U.K.
6 Singapore Eye Research Institute, Singapore 169857, Singapore; Department of Pharmacy & Pharmaceutical Sciences, National University of Singapore, Singapore 117543, Singapore; Ophthalmology and Visual Sciences Academic Clinical Program, Duke-NUS Graduate Medical School, Singapore 169857, Singapore
* Correspondence: Anish.parmar@liverpool.ac.uk (A.P); I.singh@liverpool.ac.uk (I.S).

Table of Contents

I: Materials 2
II: Equipment Used for the Analysis and Purification of Compounds 2
III: Synthesis & Analysis of Dimeric Amino Acid Building Blocks (HRMS, ^{1}H NMR, ^{13}C NMR) 2
IV: Synthesis of Alloc-Protected Amino Acid Building Blocks 11
V: Total Synthesis of Teixobactin Analogues via Dimer Approach 14
VI: Total Synthesis of Teixobactin Analogues via an Alloc-Protected Strategy 16
VII: HPLC/Mass Analysis for the Molecules 1 – 10 17
VIII: MIC Testing (Screening) 28

I: Materials

All amino acids, 1-[Bis(dimethylamino)methylene]-1H-1,2,3 triazolo[4,5-b]pyridinium3-oxidhexafluorophosphate (HATU), Phenylsilane ($PhSiH_3$), Diisopropylethylamine (DIPEA), Tritylchloride 4-(Dimethylamino)pyridine(DMAP), Tetrakis(triphenylphosphine)palladium(0) $Pd(PPh_3)_4$, Ethyl cyano(hydroxyimino)acetate (Oxyma Pure), Diisopropylcarbodiimide (DIC) and Triisopropylsilane (TIS) were purchased from Fluorochem, UK. Dimethylformamide (DMF) peptide synthesis grade was purchased from Rathburn chemicals. Triethylamine, Diethyl ether (Et_2O), Dimethylsulfoxide (DMSO), Dichloromethane (DCM), Formic acid 98-100% purity, Water (HPLC grade) and Acetonitrile (HPLC grade) were purchased from Fisher Scientific. 2-Chlorotrityl Chloride resin (manufacturer's loading: 1.60 mmol Cl^-/g) was purchased from Iris Biotech GmbH. All chemicals were used without further purification.

II: Equipment Used for the Analysis and Purification of Compounds

Compounds were analysed on a Thermo Scientific Dionex Ultimate 3000 RP-HPLC equipped with a Phenomenex Gemini NX C18 110 Å (150 x 4.6 mm) column using the following buffer systems: A: 0.1% HCOOH in water. B: ACN using a flow rate of 1 mL/min. The column was flushed with 95% A for 5 min prior to an injection and was flushed for 5 min with 95% B and 5% A after the run was finished.

Peptides were purified using the same gradient as mentioned above, on a Biotage® Isolera one flash purification system with a flow rate of 25 mL/min and monitored at 214 nm.

HRMS spectra were recorded on two machines a Thermo Scientific Q Exactive Plus Orbitrap Mass Spectrometer and Agilent QTOF 7200 Mass Spectrometer in the positive ion mode. NMR spectra were recorded at 27 °C on a Bruker Avance III HD 500 MHz spectrometer equipped with a room-temperature broadband probe.

III: Synthesis & Analysis of Dimeric Amino Acid Building Blocks (HRMS, ^{1}H NMR, ^{13}C NMR)

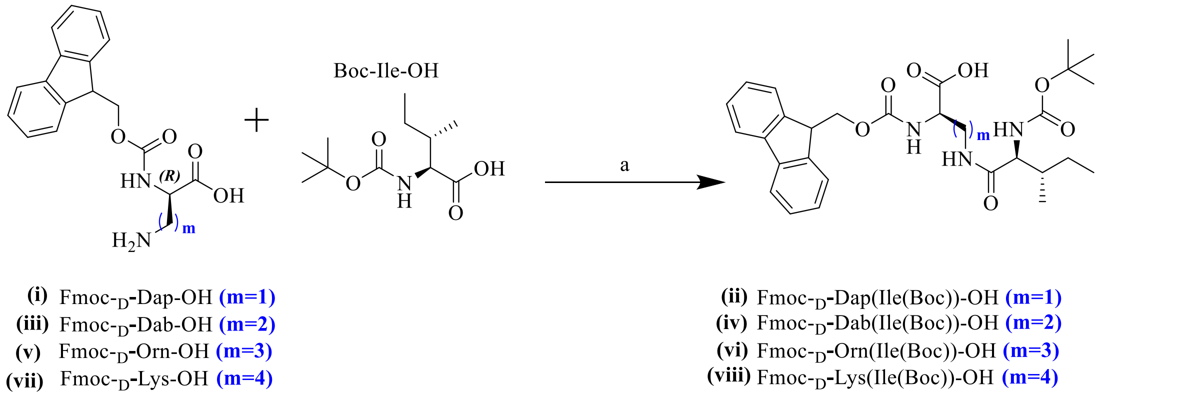

Scheme S1: Synthesis of dimeric amino acid building blocks (ii, iv, vi, viii). (a) HATU, DIPEA, DMF, 1 hr.

General procedure for the synthesis of dimeric amino acid building blocks (ii, iv, vi, viii): Fmoc-protected amino acids (Fmoc-D-Dap-OH (i), Fmoc-D-Dab-OH (iii), Fmoc-D-Orn-OH (v), or Fmoc-D-Lys-OH (vii); 1.0 eq.) were dissolved in DMF, followed by the addition of HATU (0.8 eq.) and DIPEA (3.0 eq.). The reaction mixture was stirred briefly to allow activation, after which Boc-Ile-OH (1.0 eq.) was added. The reaction was stirred at rt. for 1 hr.

The reaction mixture was then quenched by the addition of an aqueous citric acid solution (0.5 M), and the aqueous phase was extracted with DCM (3 ×). The combined organic layers were dried over anhydrous Na_2SO_4, filtered, and concentrated under reduced pressure. The crude products were purified by silica gel column chromatography using DCM/MeOH as the eluent. Isolated yields varied and typically ranged from 40–80%.

Number	Name	Chemical formula	Mass Calcd $[M+H]^+$ (Da)	Mass Obsd (Da)
**ii**	Fmoc-D-Dap-(Ile-Boc)-OH	$C_{29}H_{37}N_3O_7$	540.2705<br>$M + Na^+ =$ 562.2534	$M + Na^+ =$ 562.2520
**iv**	Fmoc-D-Dab-(Ile-Boc)-OH	$C_{30}H_{39}N_3O_7$	554.2861	554.2881
**vi**	Fmoc-D-Orn-(Ile-Boc)-OH	$C_{31}H_{41}N_3O_7$	568.6903	568.3015
**viii**	Fmoc-D-Lys-(Ile-Boc)-OH	$C_{32}H_{43}N_3O_7$	582.3174	582.3178

Table S1: Compound number, chemical formula, exact mass and mass found for compounds **ii - viii**

Compound ii (Fmoc-D-Dap-(Ile-Boc)-OH)

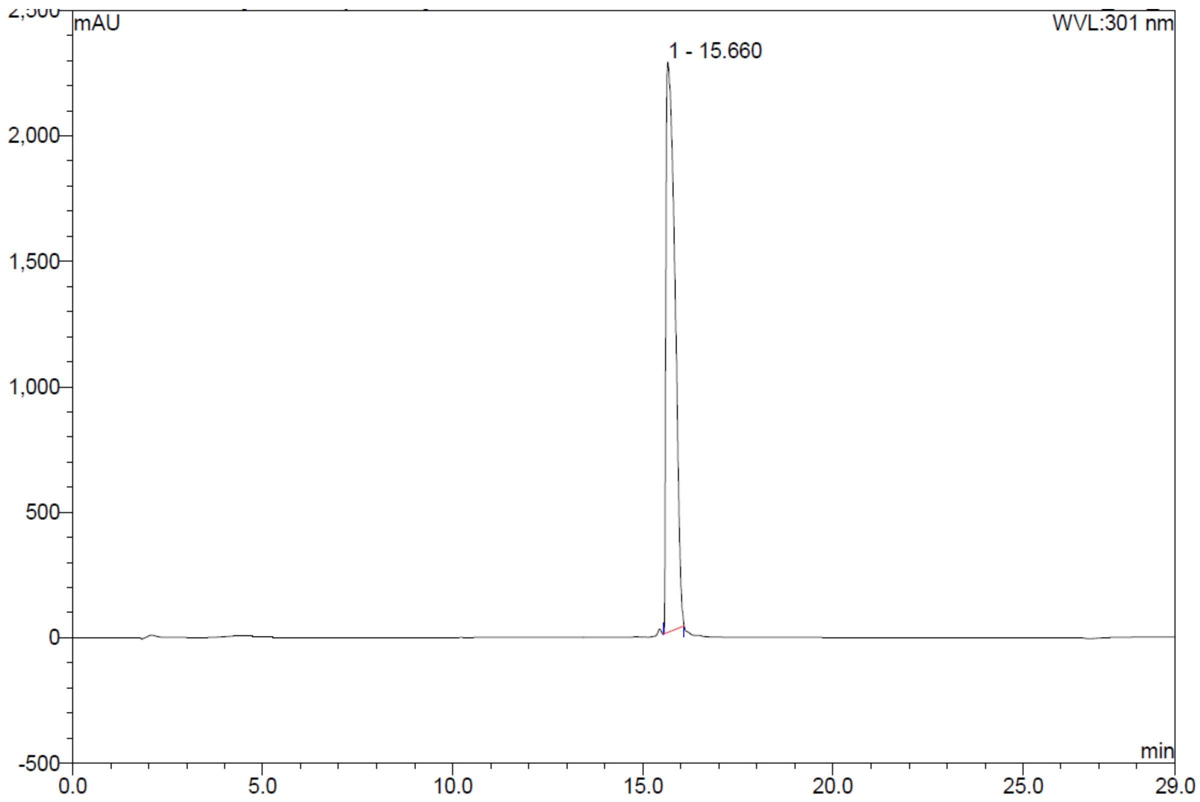

Figure S1: HPLC trace of purified dimer **ii** (gradient: 5-95% ACN in 25min using A: 0.1% HCOOH in water, B: ACN)

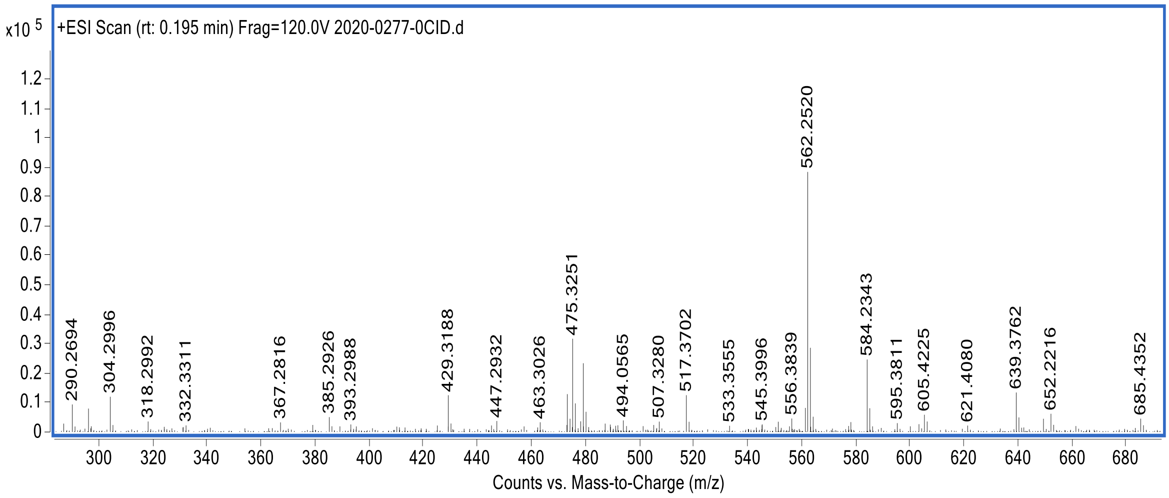

Figure S2: HRMS of purified dimer **ii**. Exact mass calcd. For $C_{29}H_{37}N_3O_7$ = 540.2705, found M + Na^+ = 562.2520

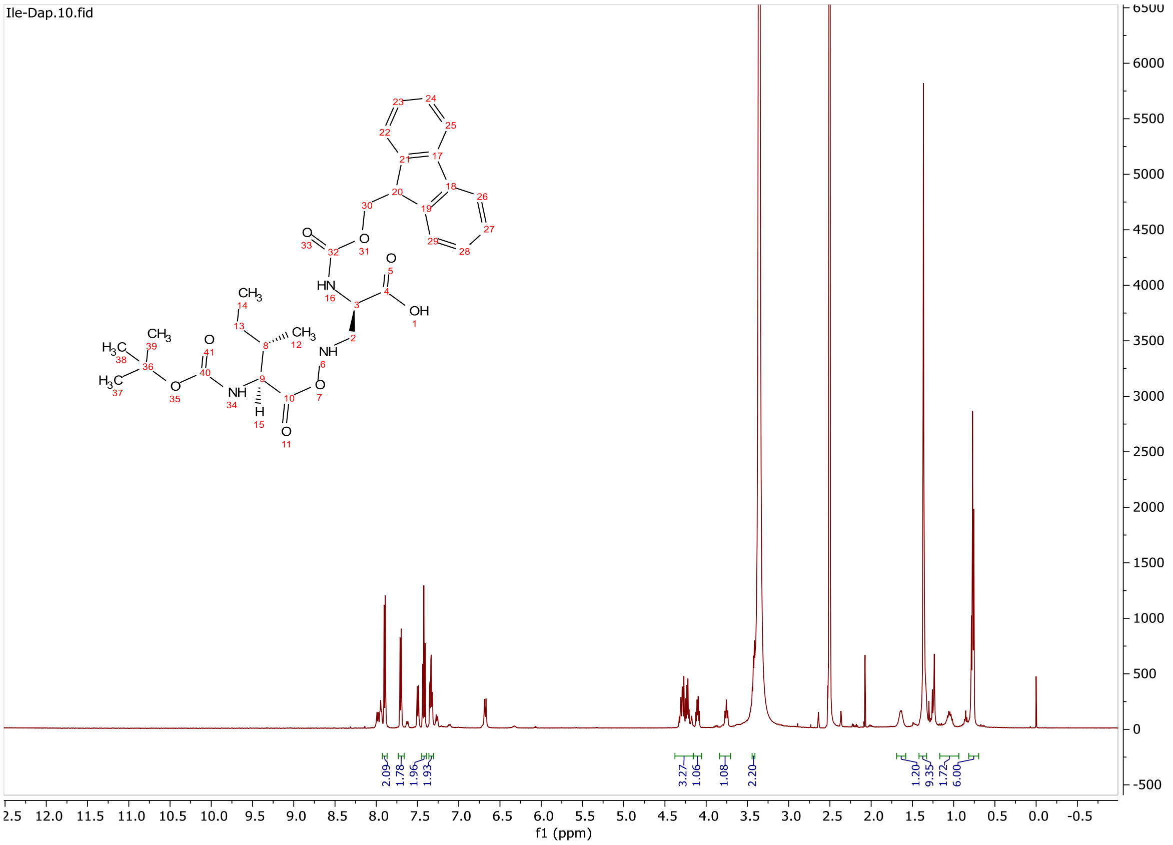

Figure S3: ^{1}H NMR (500 MHz, DMSO-d_6) at 27 °C, for **ii**. ^{1}H NMR (500 MHz, DMSO-d_6), 0.77 (t, 6H, J = 7.72 Hz, 2CH_3 of the isoleucine side chain), 1.05 (m, 2H, J = 8.12 Hz, CHCH(CH_3)C$\underline{H_2}$$CH_3$), 1.37 (s, 9H, 3$CH_3$ of the Boc), 1.65 (d, 1H, J = 6.21 Hz, CHC$\underline{H}$(CH_3)CH_2CH_3), 3.42 (d, 2H, J = 6.60 Hz, NHC$\underline{H_2}$$CH_2$NH), 3.76 (t, 1H, J = 7.95 Hz, C$\underline{H}$CH(CH_3)CH_2CH_3), 4.10 (q, 1H, J = 6.97 Hz, NHCH_2C$\underline{H}$NH), 4.40-4.17 (m , 3H, Overlap of CH and CH_2 of the Fmoc), 7.34 (t, 1H, J = 7.80 Hz, CH of the Fmoc ring at β position), 7.42 (t, 2H, J = 6.40 Hz, CH of the Fmoc ring at β position), 7.71 (d, 1H, CH of the Fmoc ring at α position), 7.90 (d, 1H, J = 7.80 Hz, CH of the Fmoc ring at α position).

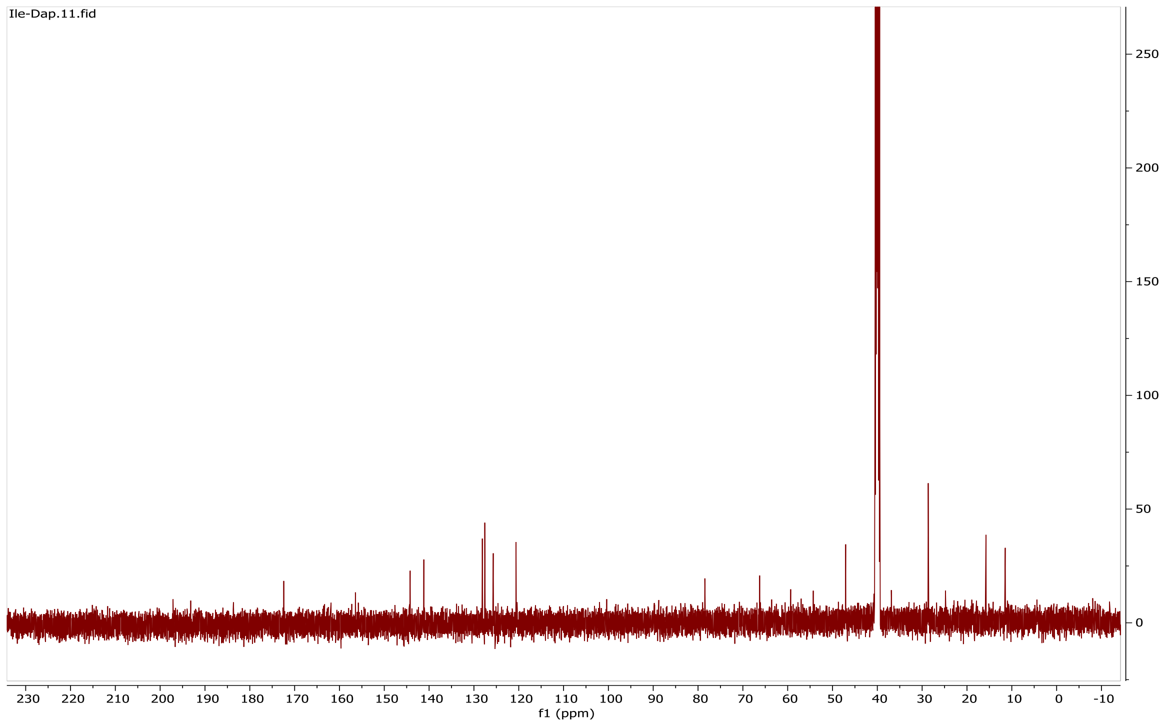

Figure S4: ^{13}C NMR (126 MHz, DMSO-d_6) at 27 °C, for **ii**. ^{13}C NMR (126 MHz, DMSO-d_6) δ, 11.46, 15.74, 24.78, 28.60, 36.89, 40.27, 47.03, 54.34, 59.28, 66.21, 120.52, 125.67, 127.57, 128.15

Compound iv Fmoc-D-Dab-(Ile-Boc)-OH

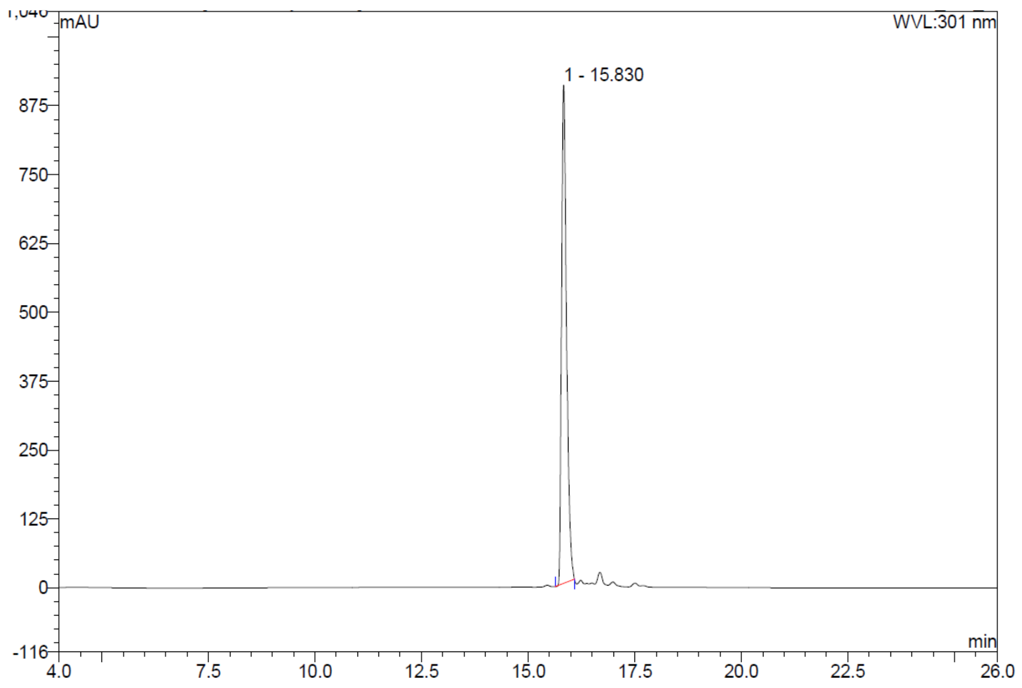

Figure S5: HPLC trace of purified dimer **iv** (gradient: 5-95% ACN in 25min using A: 0.1% HCOOH in water, B: ACN)

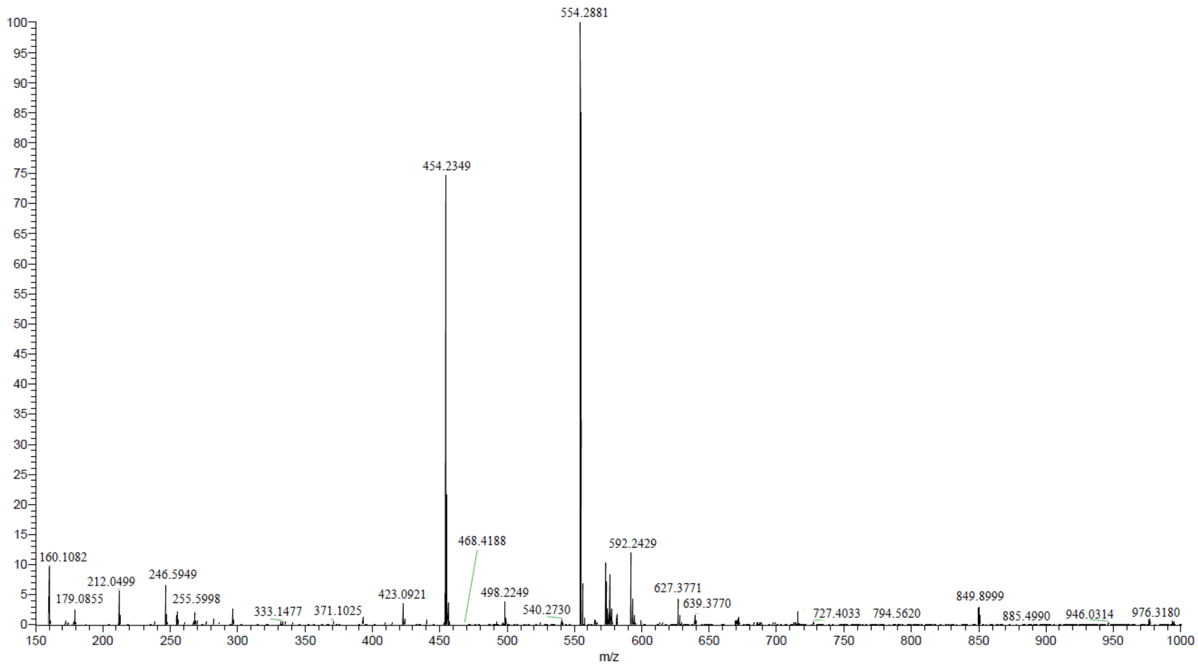

Figure S6: HRMS of purified dimer **iv**. Exact mass calcd. for $C_{30}H_{39}N_3O_7$= 554.2861, found M + H^+ = 554.2881

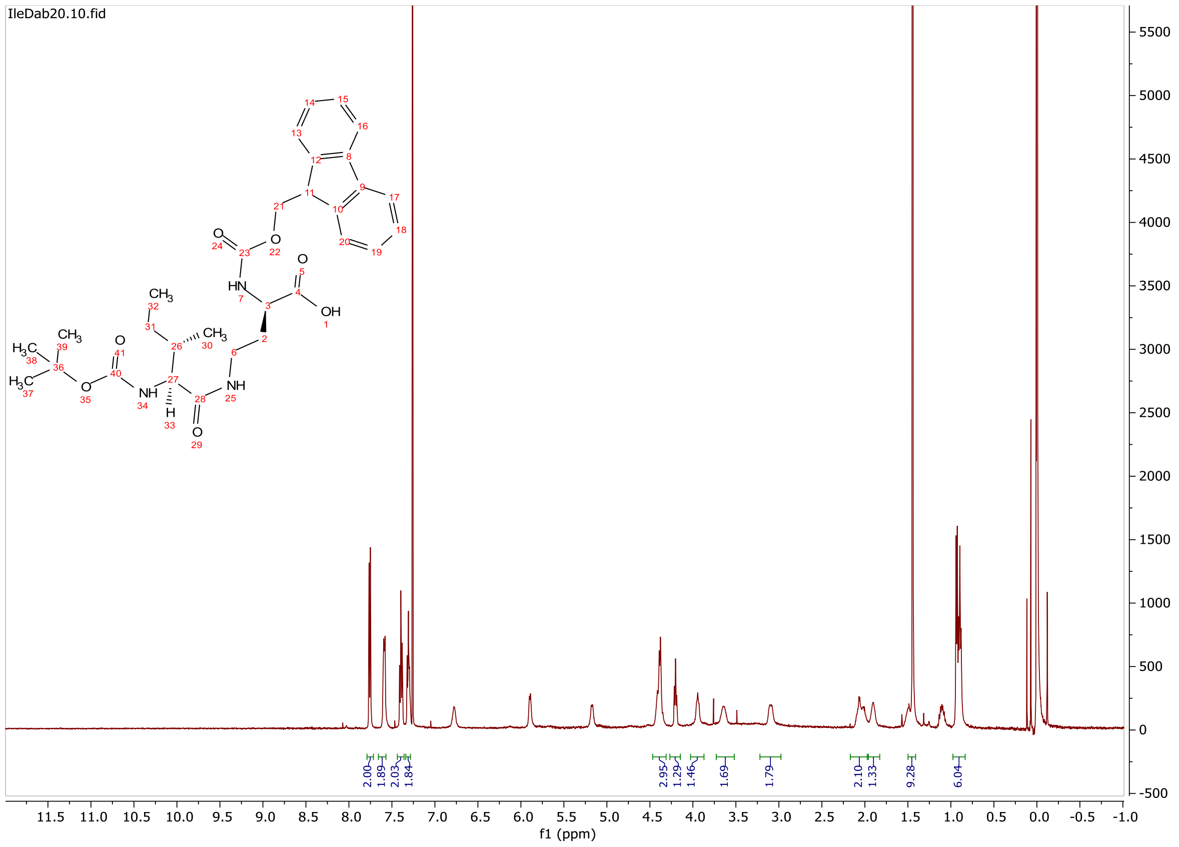

Figure S7: ^1H NMR (500 MHz, $CDCl_3$) at 27 °C, for **iv.** ^1H NMR (500 MHz, $CDCl_3$), 0.92 (m, 6H, J = 5.63 Hz $2CH_3$ of isoleucine side chain), 1.45 (s, 9H, $2CH_3$ of the Boc), 2.15-1.85(m, 3H, Overlap of CH and CH_2 for, CHC<u>H</u>(CH_3) CH_2CH_3), 3.09 (s, 2H, $NHCH_2C\underline{H_2}CH$), 3.63 (s, 2H, $NHC\underline{H_2}CH_2CH$), 3.95 (t, 1H, J = 7.92 Hz, C<u>H</u>CH(CH_3)CH_2CH_3), 4.20 (t, 1H, J = 7.92 Hz, CH_2C<u>H</u>-Fmoc), 4.46-4.31 (m, 3H, Overlap between CH and CH_2, CH_2 for $C\underline{H_2}$CH-Fmoc, and CH for C<u>H</u>(CH_2)$_2$), 7.31 (m, 1H, J = 7.32 Hz, CH of the Fmoc ring at β position), 7.39 (d, 1H, J = 7.92 Hz, CH of the Fmoc ring at β position), 7.59 (d, 1H, J = 7.92 Hz, CH of the Fmoc ring at α position), 7.76 (d, 1H, J = 7.12 Hz, CH of the Fmoc ring at α position)

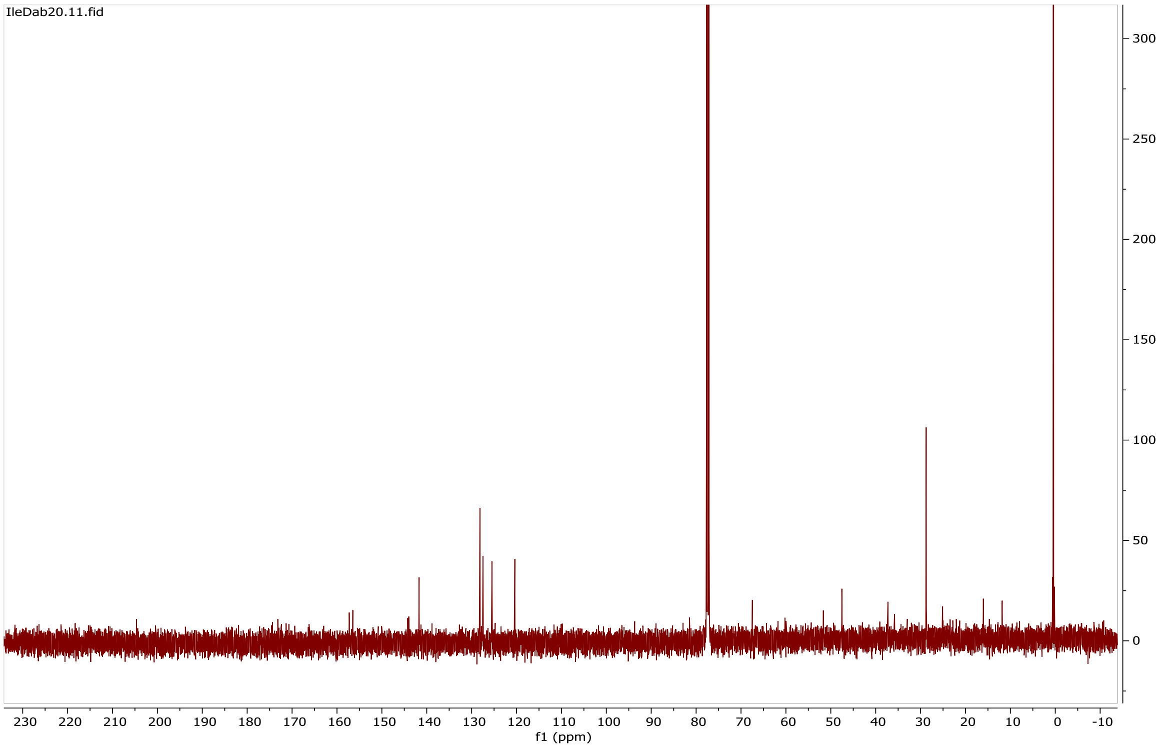

Figure S8: ^{13}C NMR (126 MHz, $CDCl_3$) at 27 °C, for **iv**. ^{13}C NMR (126 MHz, $CDCl_3$) δ 11.83,15.63, 23.60, 24.67, 24.67, 24.76, 28.75, 35.39, 35.46, 37.05,47.54, 59.75, 120.39, 125.50, 127.48, 128.15

Compound vi Fmoc-D-Orn-(Ile-Boc)-OH

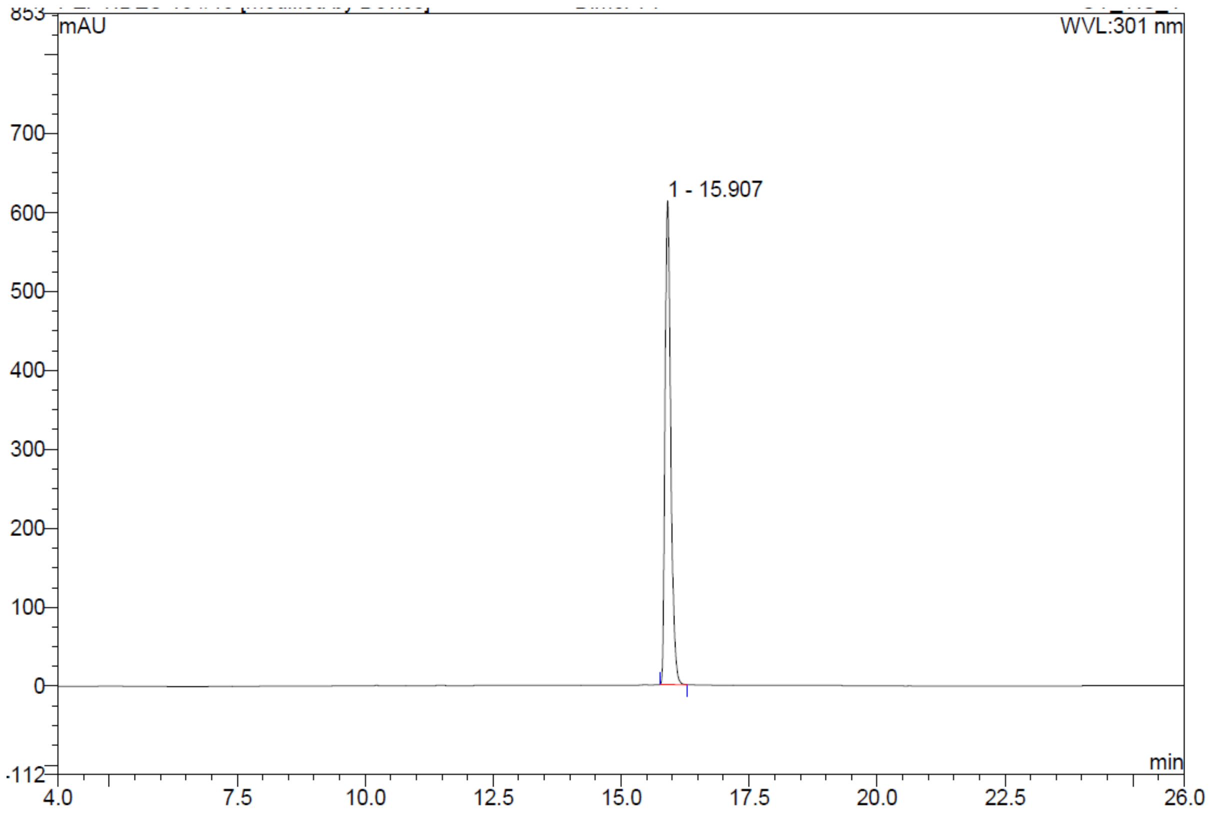

Figure S9: HPLC trace of purified dimer **vi** (gradient: 5-95% ACN in 25min using A: 0.1% HCOOH in water, B: ACN)

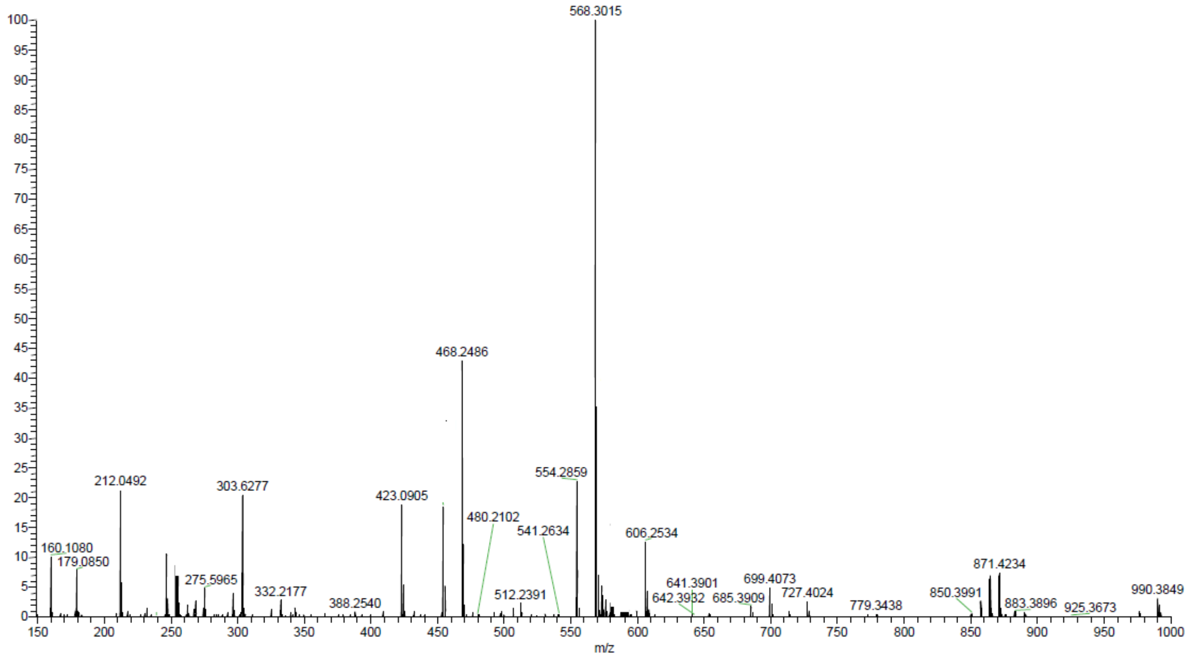

Figure S10: HRMS of purified dimer **vi**. Exact mass calcd. for $C_{31}H_{41}N_3O_7$= 568.6903, found M + H^+ = 568.3015

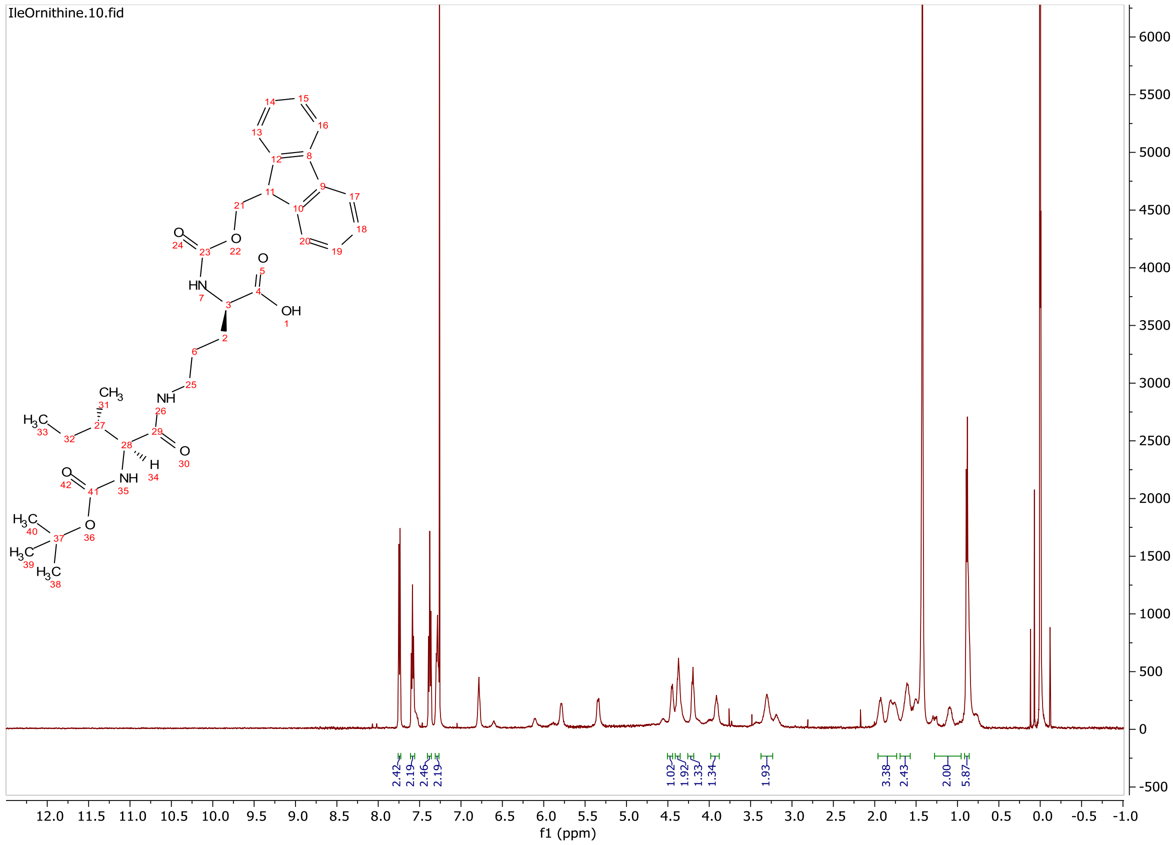

Figure S11: ^{1}H NMR (500 MHz, $CDCl_3$) at 27 °C, for **vi**. ^{1}H NMR (500 MHz, $CDCl_3$), 0.98 (d, 6H, J = 6.75 Hz, $2CH_3$ of the isoleucine side chain), 1.10 (s, 2H, $CHCH(CH_3)$ CH_2CH_3), 1.43 (s,9H, $3CH_3$ of the Boc), 1.61 (m, 2H, J = 6.08 Hz, $COOHCHCH_2C\underline{H_2}CH_2$), 1.98-1.67 (An overlap between CH and CH_2, CH for $CHC\underline{H}(CH_3)CH_2CH_3$ And CH_2 for $COOHCHC\underline{H_2}(CH_2)_2$), 3.32 (s, 2H, $CH(CH_2)_2C\underline{H_2}$), 3.92 (s, 1H, $C\underline{H}CH(CH_3)CH_2CH_3$), 4.20 (d, 1H, J= 7.43 Hz, $CH_2C\underline{H}$-Fmoc), 4.36 (d, 2H, J = 6.87, $C\underline{H_2}CH$-Fmoc), 4.45 (d, 2H, J = 7.92 Hz, $COOHC\underline{H}(CH_2)_3$), 7.28 (t, 1H, J = 7.63 Hz, CH of the Fmoc ring at β position), 7.38 (t, 1H, J = 8.16, CH of the Fmoc ring at β position), 7.59 (t, 1H, J = 8.16 Hz, CH of the Fmoc ring at α position), 7.74 (d, 1H, J = 8.12 Hz, CH of the Fmoc ring at α position)

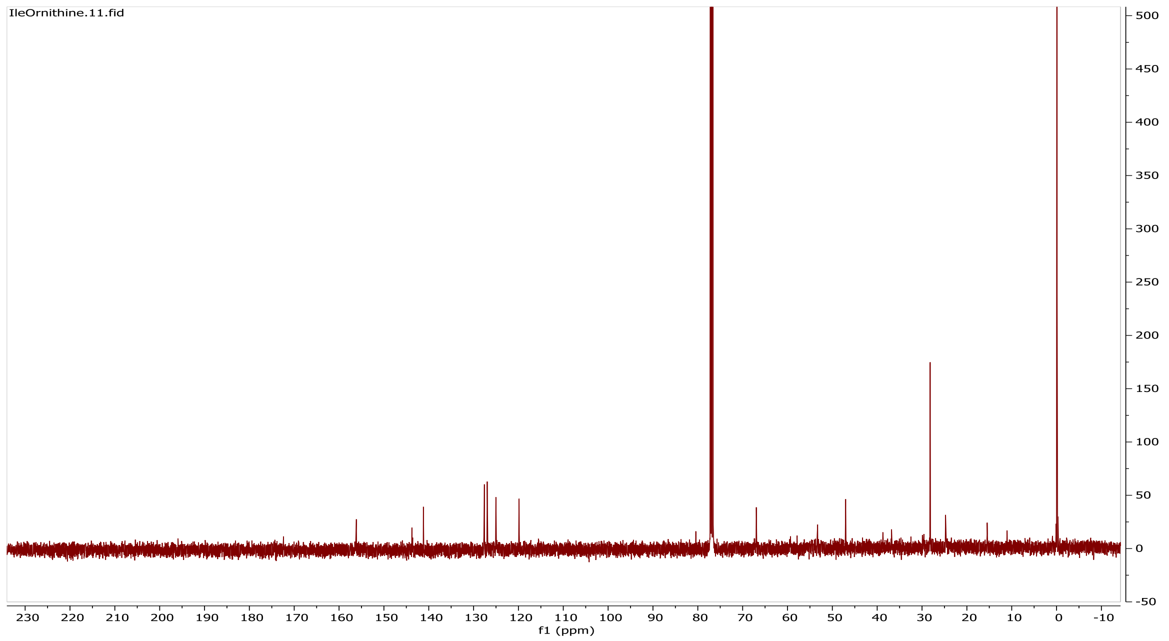

Figure S12: ^{13}C NMR (126 MHz, $CDCl_3$) at 27 °C, for **vi**. ^{13}C NMR (126 MHz, $CDCl_3$) δ, 11.21, 15.46, 24.75, 24.87, 28.18, 29.64, 29.76, 38.85, 47.03, 53.29, 59.48, 67.10, 119.93, 125.04, 127.09, 127.73

Compound viii Fmoc-D-Lys-(Ile-Boc)-OH

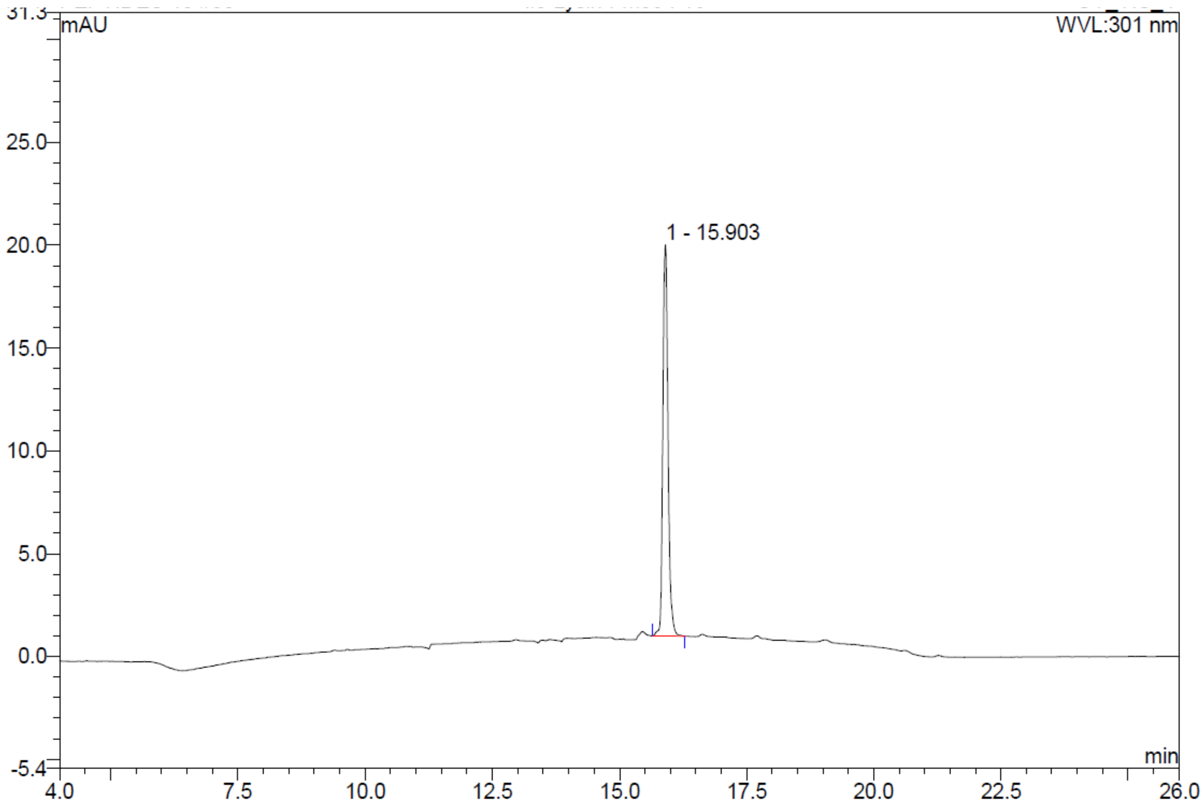

Figure S13: HPLC trace of purified dimer **viii** (gradient: 5-95% ACN in 25min using A: 0.1% HCOOH in water, B: ACN)

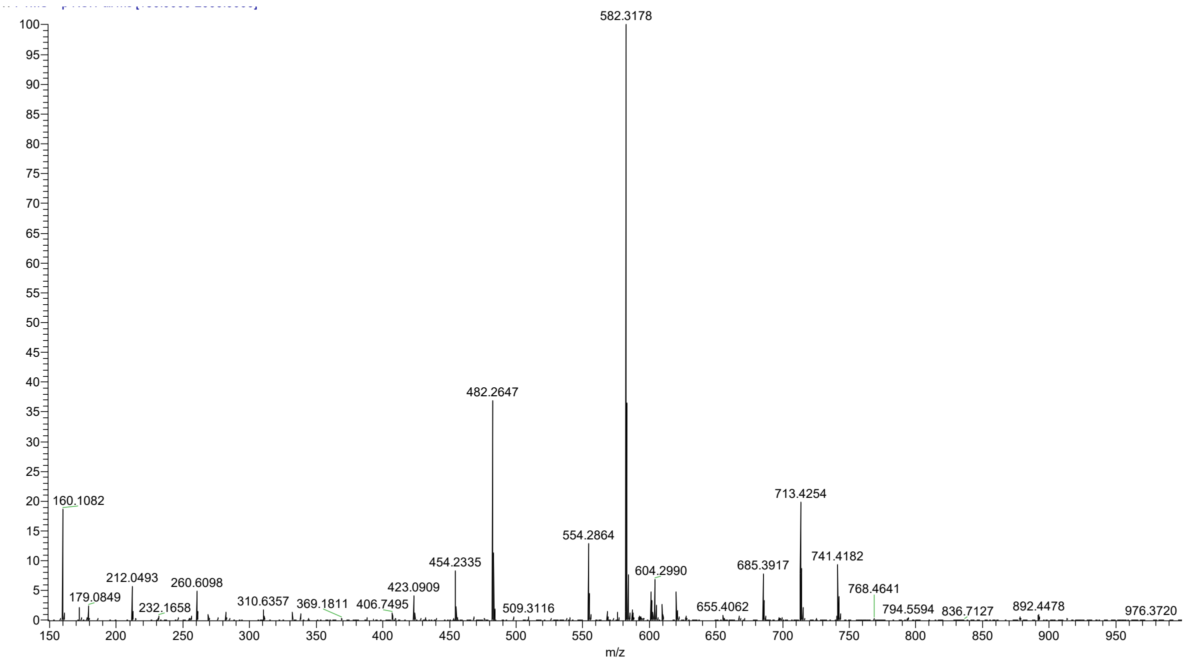

Figure S14: HRMS of purified dimer **viii**. Exact mass calcd. for $C_{32}H_{43}N_3O_7$ = 582.3174, found $[M + H]^+$ = 582.3178

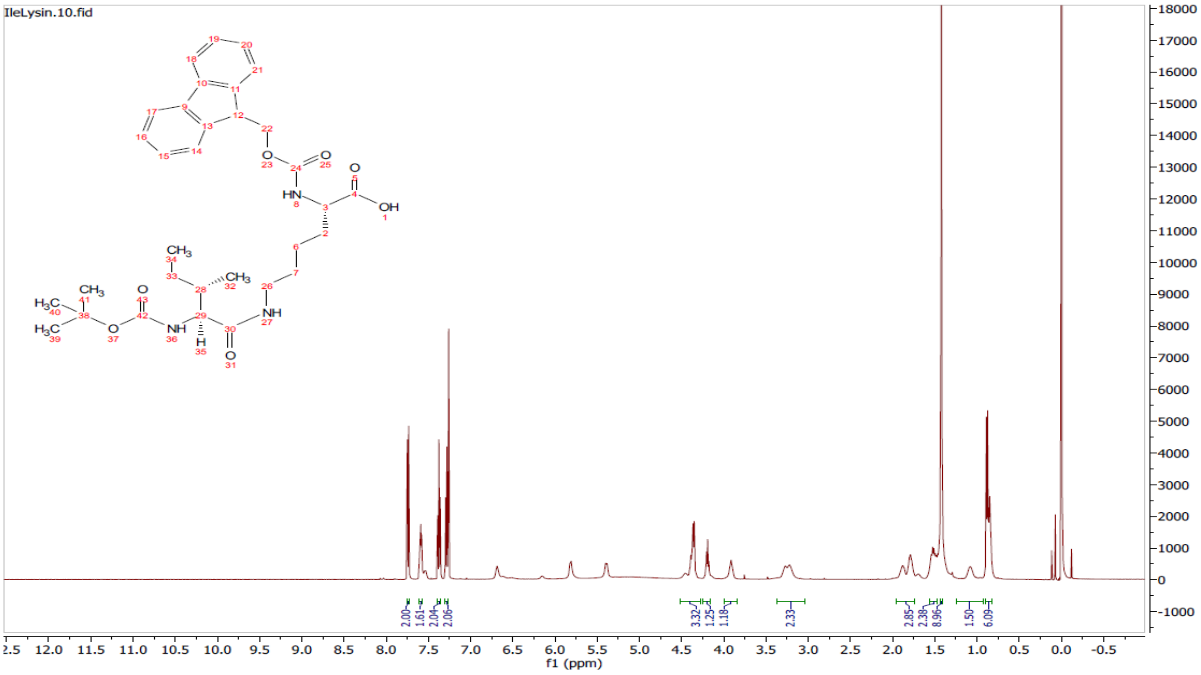

Figure S15: ^{1}H NMR (500 MHz, $CDCl_3$) at 27 °C, for **viii.** ^{1}H NMR (500 MHz, $CDCl_3$), 0.88 (t, 6H, J = 6.31 Hz, $2CH_3$ of isoleucine side chain), 1.08 (s, 2H, $NH(CH_2)_2C\underline{H_2}CH$), 1.42 (s, 9H, $3CH_3$ of the Boc), 1.52 (m, 2H, J = 7.82 Hz, $NH(CH2)2C\underline{H_2}CH_2$), 1.95-1.73 (m, 3H, overlap of CH and CH_2, for $NHCHC\underline{H}(CH_3)C\underline{H_2}CH_3$), 3.24 (d, 2H, J = 22.37 Hz, $NHC\underline{H_2}(CH_2)_3$), 3.90 (t, J = 8.82 Hz, $C\underline{H}CH(CH_3)CH_2CH_3$), 4.19 (t, 1H, J = 6.99 Hz, $CH_2C\underline{H}$-Fmoc), 4.37 (m, 3H, Overlap for CH and CH_2, CH_2 for $C\underline{H_2}$CH-Fmoc, and CH for NH-$C\underline{H}(CH_2)_4$), 7.28 (t, 1H, J = 8.30 Hz, CH of the Fmoc ring at β position), 7.37 (t, 1H, J = 9.38 Hz, CH of the Fmoc ring at β position), 7.59 (t, 1H, J = 5.78 Hz, CH of the Fmoc ring at α position), 7.74 (d, 1H, J = 7.23 Hz, CH of the Fmoc ring at α position)

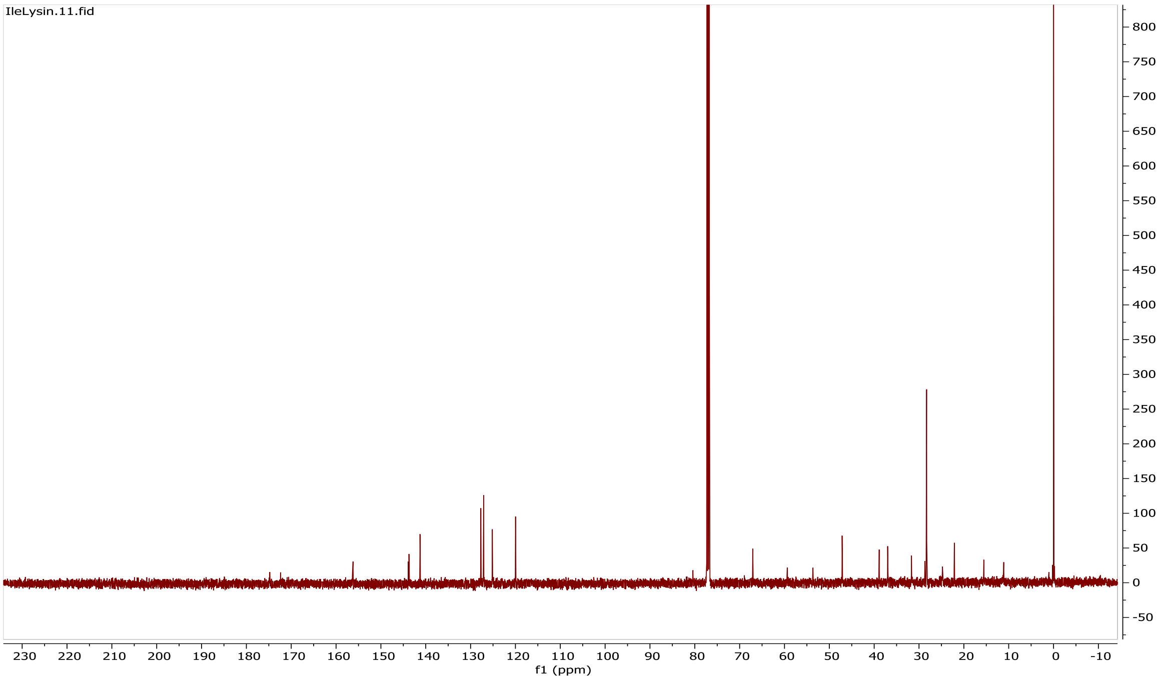

Figure S16: ^{13}C NMR (126 MHz, $CDCl_3$) at 27 °C, for **viii.** ^{13}C NMR (126 MHz, $CDCl_3$) δ, 11.18, 15.54, 24.78, 28.31, 28.68, 31.68, 36.99, 38.88, 47.15, 53.77, 59.39, 67.08, 119.97, 125.16, 127.08, 127.71

IV: Synthesis of Alloc-protected Amino Acid Building Blocks

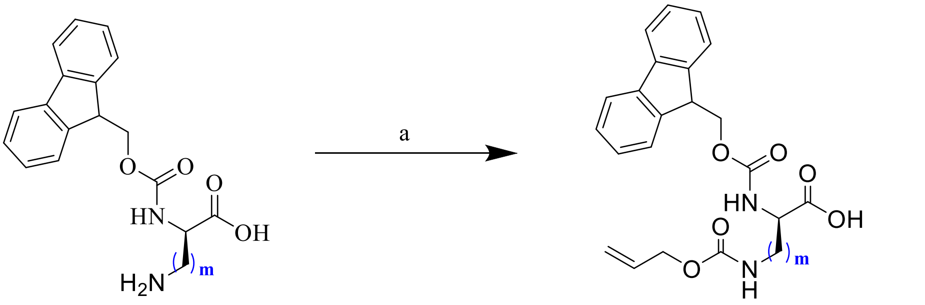

(i) Fmoc-D-Dap-OH **(m=1)**
(iii) Fmoc-D-Dab-OH **(m=2)**
(v) Fmoc-D-Orn-OH **(m=3)**
(vii) Fmoc-D-Lys-OH **(m=4)**

(ix) Fmoc-D-Dap(Alloc)-OH **(m=1)**
(x) Fmoc-D-Dab(Alloc)-OH **(m=2)**
(xi) Fmoc-D-Orn(Alloc)-OH **(m=3)**
(xii) Fmoc-D-Lys(Alloc)-OH **(m=4)**

Scheme S2. Synthesis of Alloc protected Amino acid building blocks (ix-xiii)

General procedure for Alloc protection of amino acids building blocks ix-xiii: Fmoc-protected amino acids (Fmoc-D-Dap-OH (i), Fmoc-D-Dab-OH (iii), Fmoc-D-Orn-OH (v), or Fmoc-D-Lys-OH (vii); 1.0 equiv) were dissolved in THF (10 mL), followed by the addition of $NaHCO_3$ (5.0 equiv). Allyl chloroformate (1.5 equiv) was added dropwise, and the reaction mixture was stirred at rt. overnight. Reaction progress was monitored by TLC. Upon completion, the reaction mixture was diluted with water (50 mL) and acidified to pH 2 using 1 M HCl. The aqueous phase was extracted with DCM (3 × 50 mL), and the combined organic layers were dried over anhydrous Na_2SO_4, filtered, and concentrated under reduced pressure. The crude products were purified by silica gel column chromatography using DCM/MeOH as the eluent to afford the corresponding Fmoc- and Alloc-protected amino acid building blocks. Isolated yields varied and typically ranged between 45-80%. Product identity was confirmed by LC-MS, and the compounds were used directly for subsequent peptide synthesis.

**Number**	**Name**	**Chemical formula**	**Mass Calcd [M-H]$^-$ (Da)**	**Mass Obsd (Da)**
**ix**	Fmoc-D-Dap(Alloc)-OH	$C_{22}H_{22}N_2O_6$	409.14	409.4
**x**	Fmoc-D-Dab(Alloc)-OH	$C_{23}H_{24}N_2O_6$	423.16	423.2
**xi**	Fmoc-D-Orn(Alloc)-OH	$C_{24}H_{26}N_2O_6$	437.17	437.3
**xii**	Fmoc-D-Lys(Alloc)-OH	$C_{25}H_{28}N_2O_6$	451.19	451.3

Table S2: Compound number, chemical formula, exact mass and mass found for compounds **ix - xii**

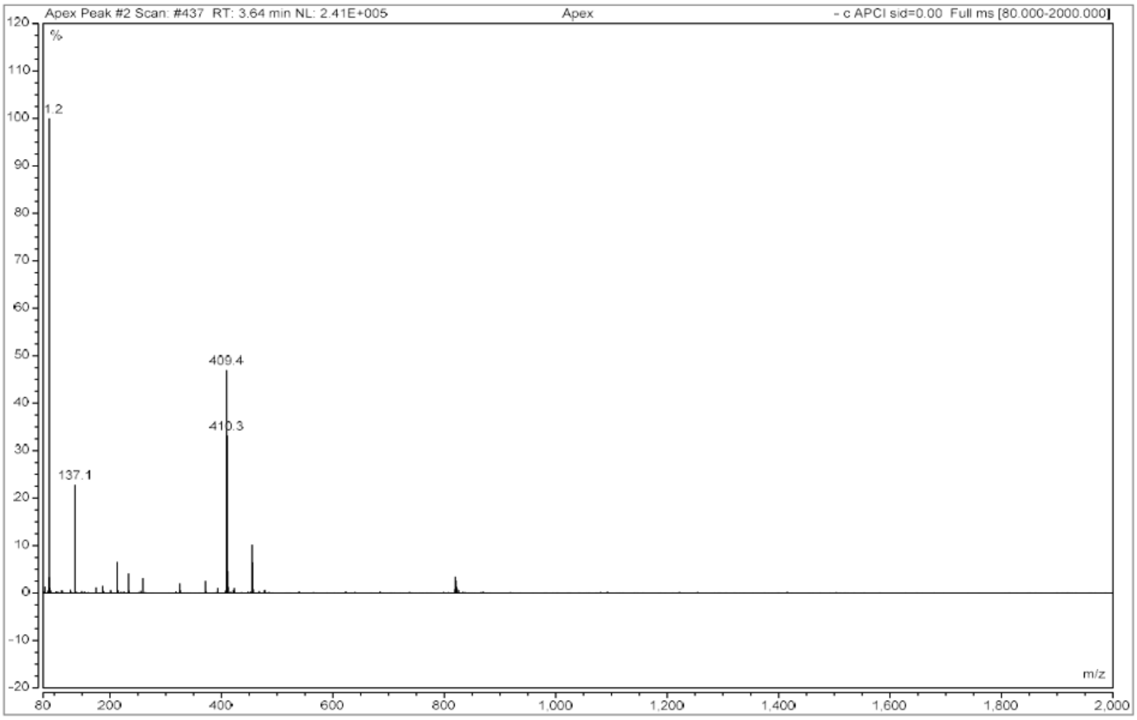

Figure S17: MS of purified building block **ix** (Fmoc-D-Dap(Alloc)-OH). Exact mass calcd. for $C_{22}H_{22}N_2O_6$= 409.14, found M-H$^-$ = 409.4

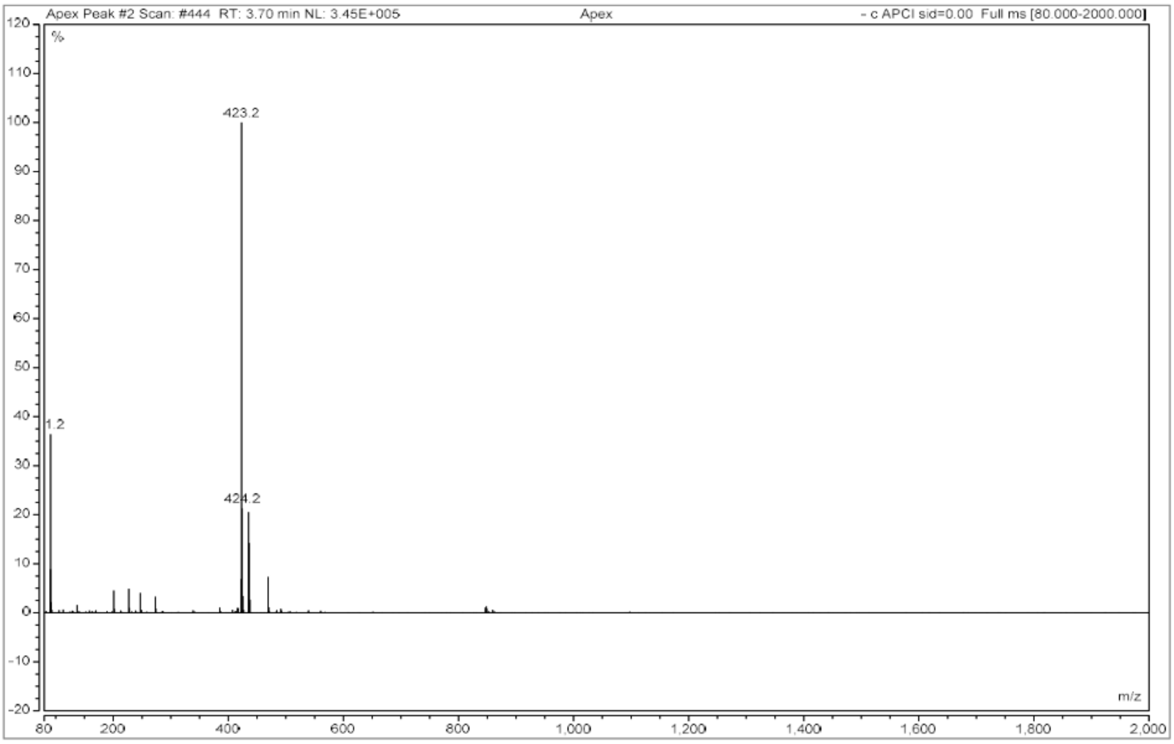

Figure S18: MS of purified building block **x** (Fmoc-D-Dab(Alloc)-OH). Exact mass calcd. for $C_{23}H_{24}N_2O_6$=423.16, found M - H^- = 423.2

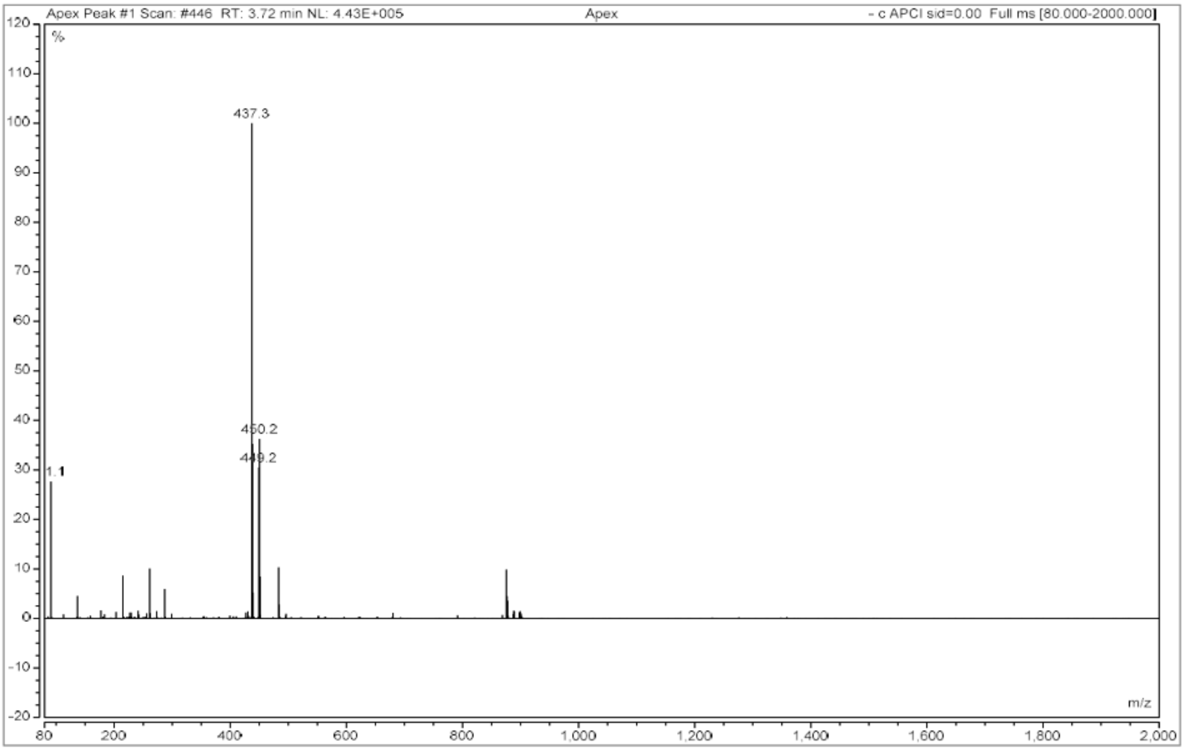

Figure S19: MS of purified building block **xi** (Fmoc-D-Orn(Alloc)-OH). Exact mass calcd. for $C_{24}H_{26}N_2O_6$=437.17, found M - H^- = 437.3

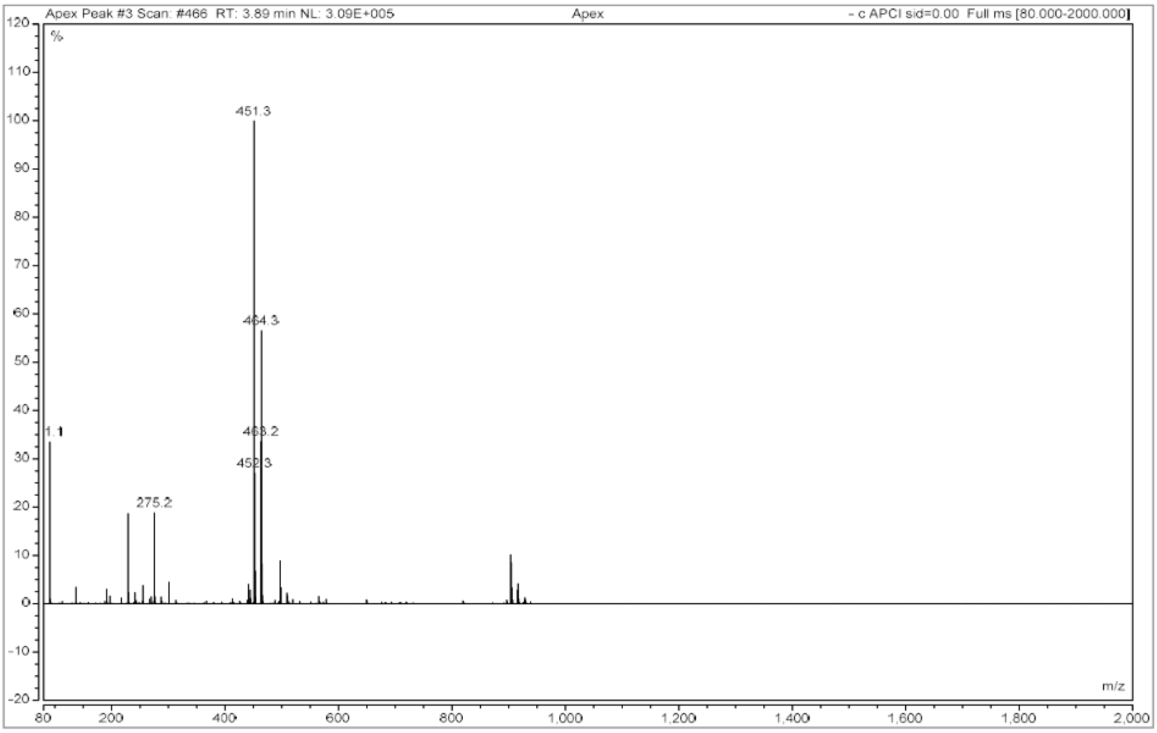

Figure S20: MS of purified building block **xii** (Fmoc-D-Lys(Alloc)-OH). Exact mass calcd. for $C_{25}H_{28}N_2O_6$=451.19, found M - H$^-$ = 451.3

V: Total Synthesis of Teixobactin Analogues via Dimer Approach

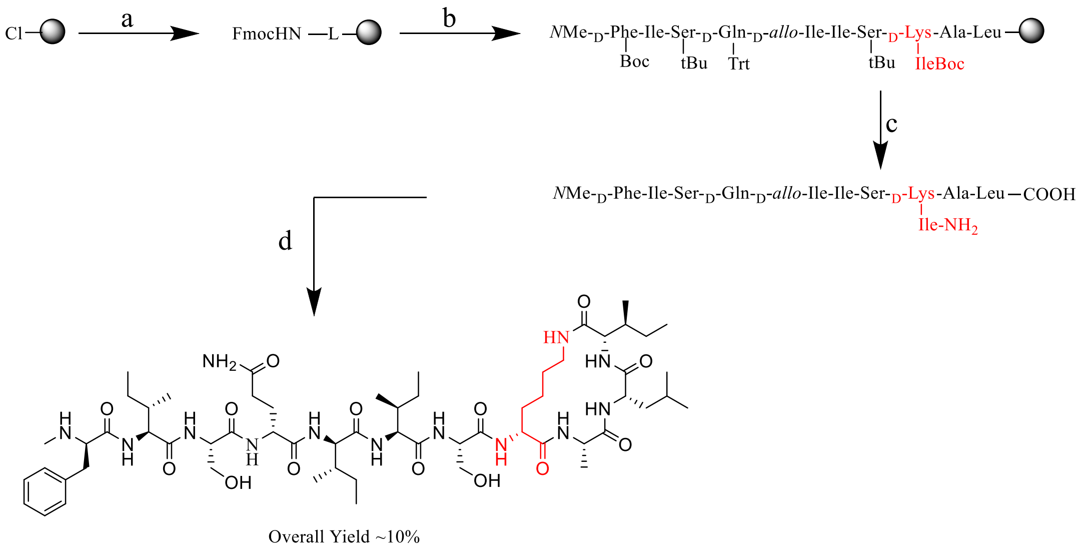

Scheme S3. Synthesis of teixobactin analogue 9 via the dimer approach. (step a) Commercially available 2-Chlorotrityl chloride resin (manufacturer's loading = 1.6 mmol/g) was swelled in DCM in a reactor. Fmoc-Leu-OH was loaded to the resin using 4 eq. AA/8 eq. DIPEA in DCM and the reactor was shaken for 3 hr. The loading of piperidine-dibenzofulvene adduct was determined by UV absorption to be 0.56 mmol/g, (179 mg resin, 0.1 mmol). Any unreacted resin was capped with MeOH:DIPEA:DCM = 1:2:7 by shaking for 1 hr. The protecting group Fmoc was removed using 20% Piperidine in DMF 2 x 10 min. (step b) The rest of the amino acids were coupled using 4 eq. AA, 4 eq. DIC/Oxyma Pure using a microwave peptide synthesiser for 10 min at 50 ºC. Deprotection was performed with microwave irradiation using 20% Piperidine for 3 min and 10 min at rt. (step c) The peptide was fully cleaved off using TFA:TIS:H_2O = 95:2.5:2.5 % by shaking for 1 hr. The acyclic peptide was precipitated using cold Et_2O (-20 °C) and centrifuging at 7000 rpm. (step d) The acyclic peptide was cyclised by adding mixture of DMF / 3 eq. HATU/10 eq. DIPEA and the cyclisation was performed for 1 hr at rt. The cyclic peptide was again precipitated using cold Et_2O (-20 °C) and centrifuging at 7000 rpm to obtain a white solid. The solid was further purified using reverse phase (C18) on a Biotage®Isolera one flash purification system. Pure fractions were pooled and lyophilised to obtain a white solid 11mg, 10% yield. Yields ranged between 8-12% for other analogues using this approach.

VI: Total Synthesis of Teixobactin Analogues via an Alloc-Protected Strategy

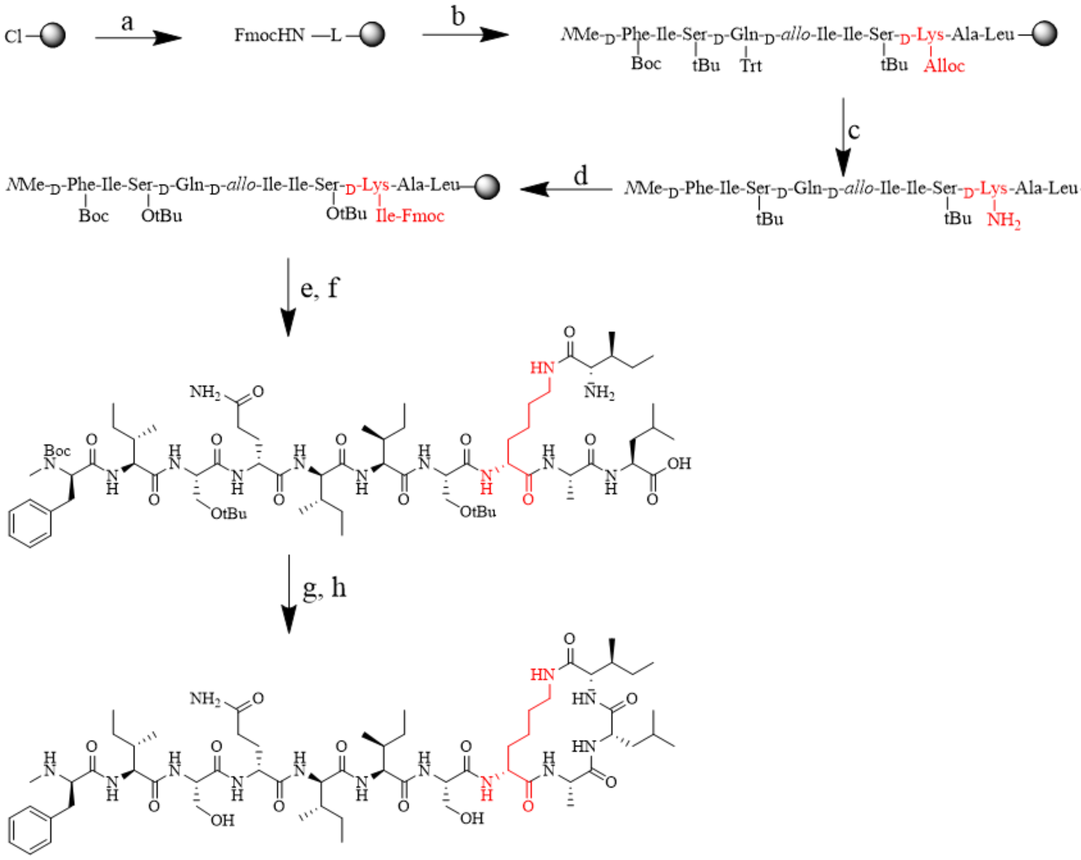

Scheme S4. Synthesis of teixobactin analogue 9 via the Alloc-protected strategy. (step a) Commercially available 2-Chlorotrityl chloride resin (manufacturer's loading = 1.6 mmol/g) was swelled in DCM in a reactor. Fmoc-Leu-OH was loaded to the resin using 4 eq. AA/8 eq. DIPEA in DCM and the reactor was shaken for 3 hr. The loading of piperidine-dibenzofulvene adduct was determined by UV absorption to be 0.56 mmol/g, (179 mg resin, 0.1 mmol). Any unreacted resin was capped with MeOH:DIPEA:DCM = 1:2:7 by shaking for 1 hr. The protecting group Fmoc was removed using 20% piperidine in DMF 2 x 10 min. (step b) The Fmoc protecting group was deprotected using 20% piperidine in DMF by shaking for 3 min, followed by draining and shaking again with 20% piperidine in DMF for 10 min. The rest of the amino acids were coupled using an automated synthesiser using 4 eq. AA, 4 eq. HATU and 8 eq. DIPEA for 1h at rt., with the same deprotection conditions mentioned earlier. (step c) The Alloc protecting group was removed using 2 eq. $Pd(PPh_3)_4$ and 24 eq. $PhSiH_3$ in dry DCM under argon for 30 min. This procedure was repeated increasing the time to 45 min and the resin was washed thoroughly with DCM and DMF to remove any leftover Pd on resin. (step d) Esterification was performed using 10 eq. of Fmoc-Ile-OH, 10 eq. DIC and 5 mol% DMAP in DCM and shaking the reaction for 1 hr. This was followed by capping the unreacted alcohol using 10% Ac_2O/DIPEA in DMF shaking for 30 min and (step e) Fmoc was removed using protocol described earlier in step (b). (step f) The peptide was cleaved from the resin without cleaving off the protecting groups of the amino acid side chains using TFA:TIS:DCM = 2:5:93 and shaking for 1 hr. (step g) The solvent was evaporated and the peptide was redissolved in DMF to which 1 eq. HATU and 10 eq. DIPEA were added and the reaction was stirred for 30 min to perform the cyclisation. (step h) The side-chain protecting groups were then cleaved off using TFA:TIS:H_2O = 95:2.5:2.5 by stirring for 2h. The peptide was precipitated using cold Et_2O (-20°C) and centrifuging at 7800 rpm to obtain a white solid. The solid was further purified using reverse phase (C18) on a Biotage®Isolera one flash purification system. Pure fractions were pooled and lyophilised to obtain a white solid 20 mg, ~18 % yield. yields ranged between 16-18% for other analogues using this approach.

Number	Name	Chemical formula	Mass Calcd $[M+H^+]$ (Da)	Mass Obsd $[M + H^+]$ (Da)
**1**	D-Dap$_8$-Leu$_{10}$-Linear-teixobactin	$C_{57}H_{97}N_{13}O_{15}$	1204.73	1204.68
**2**	D-Dab$_8$-Leu$_{10}$-Linear-teixobactin	$C_{57}H_{96}N_{13}O_{15}$	1218.75	1218.68
**3**	D-Orn$_8$-Leu$_{10}$-Linear-teixobactin	$C_{59}H_{101}N_{13}O_{15}$	1232.76	1232.63
**4**	D-Lys$_8$-Leu$_{10}$-Linear-teixobactin	$C_{60}H_{103}N_{13}O_{15}$	1246.78	1245.79
**5**	D-Ser$_8$-Leu$_{10}$-Linear-teixobactin	$C_{57}H_{96}N_{12}O_{16}$	1205.71	1204.79
**6**	D-Dap$_8$-Leu$_{10}$-teixobactin	$C_{57}H_{95}N_{13}O_{14}$	1186.72	1186.77
**7**	D-Dab$_8$-Leu$_{10}$-teixobactin	$C_{58}H_{97}N_{13}O_{14}$	1200.72	1200.73
**8**	D-Orn$_8$-Leu$_{10}$-teixobactin	$C_{59}H_{99}N_{13}O_{14}$	1214.75	1214.71
**9**	D-Lys$_8$-Leu$_{10}$-teixobactin	$C_{60}H_{101}N_{13}O_{14}$	1228.77	1228.75
**10**	D-Ser$_8$-Leu$_{10}$ -teixobactin	$C_{57}H_{94}N_{12}O_{15}$	1187.90	1187.10

Table S3: Compound number, name, chemical formula, exact mass and mass found for compounds **1 – 10.**

VII: HPLC/Mass Analysis for Analogues 1 – 10

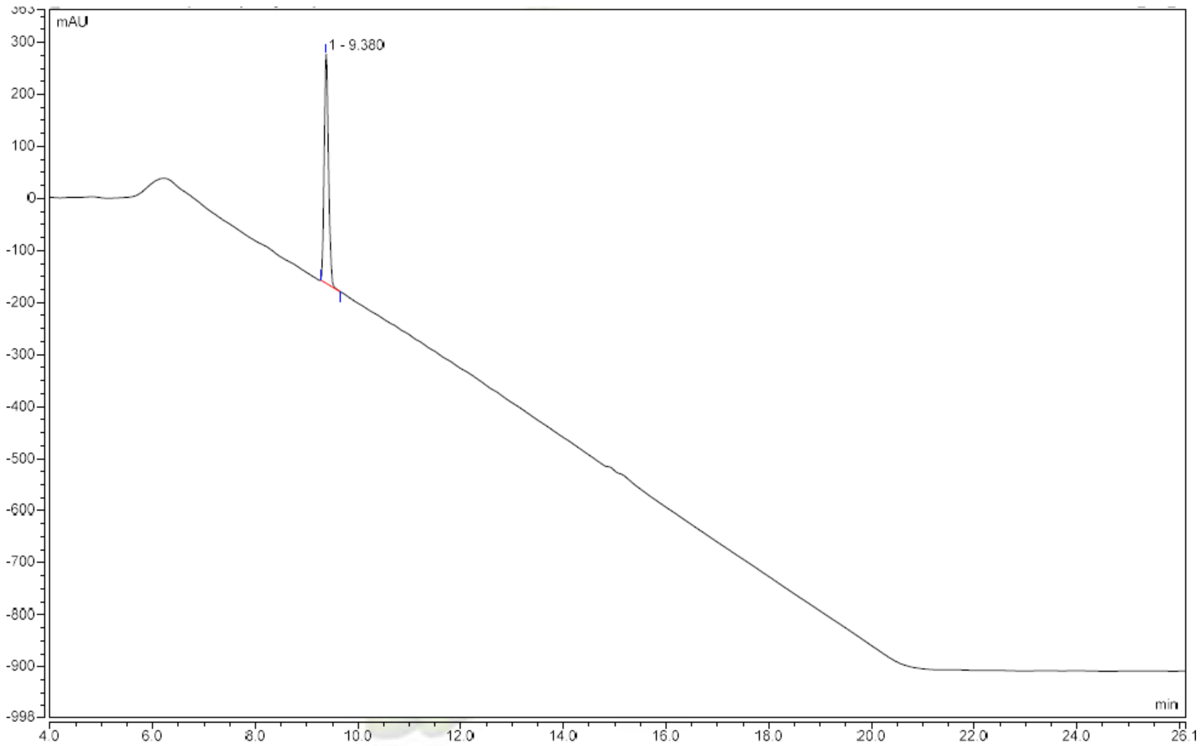

Figure S21: HPLC trace of purified teixobactin analogue **1** (gradient: 5-95% ACN in 25min using A: 0.1% HCOOH in water, B: ACN)

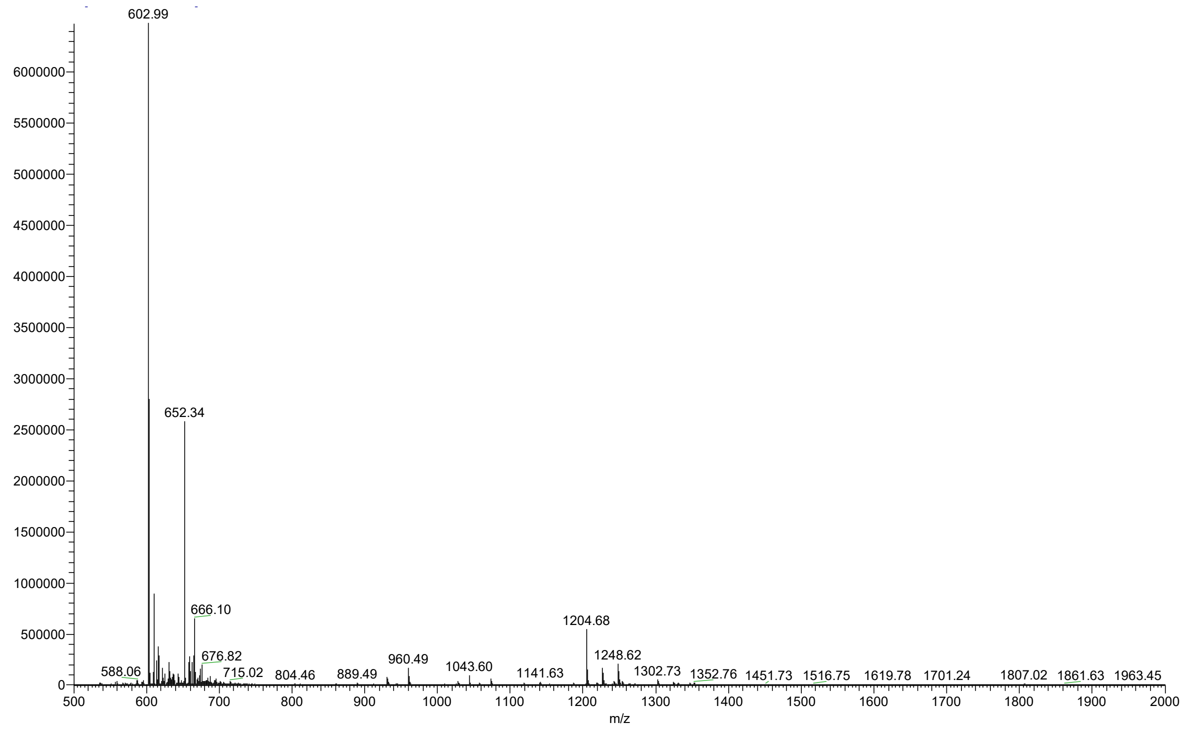

Figure S22: ESI-MS of purified teixobactin analogue **1**. Exact mass calcd. for $C_{57}H_{97}N_{13}O_{15}$ = 1204.73, found M + H$^+$ = 1204.68, M/2 + H$^+$ = 602.99

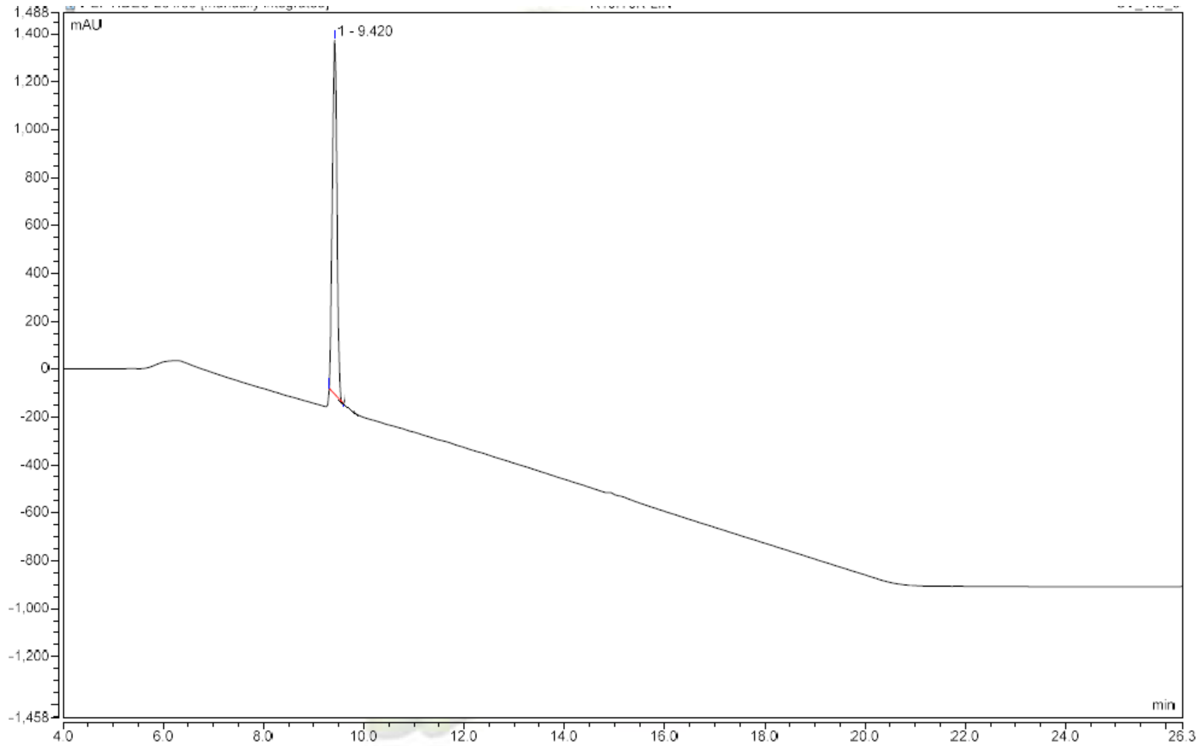

Figure S23: HPLC trace of purified teixobactin analogue **2** (gradient: 5-95% ACN in 25min using A: 0.1% HCOOH in water, B: ACN)

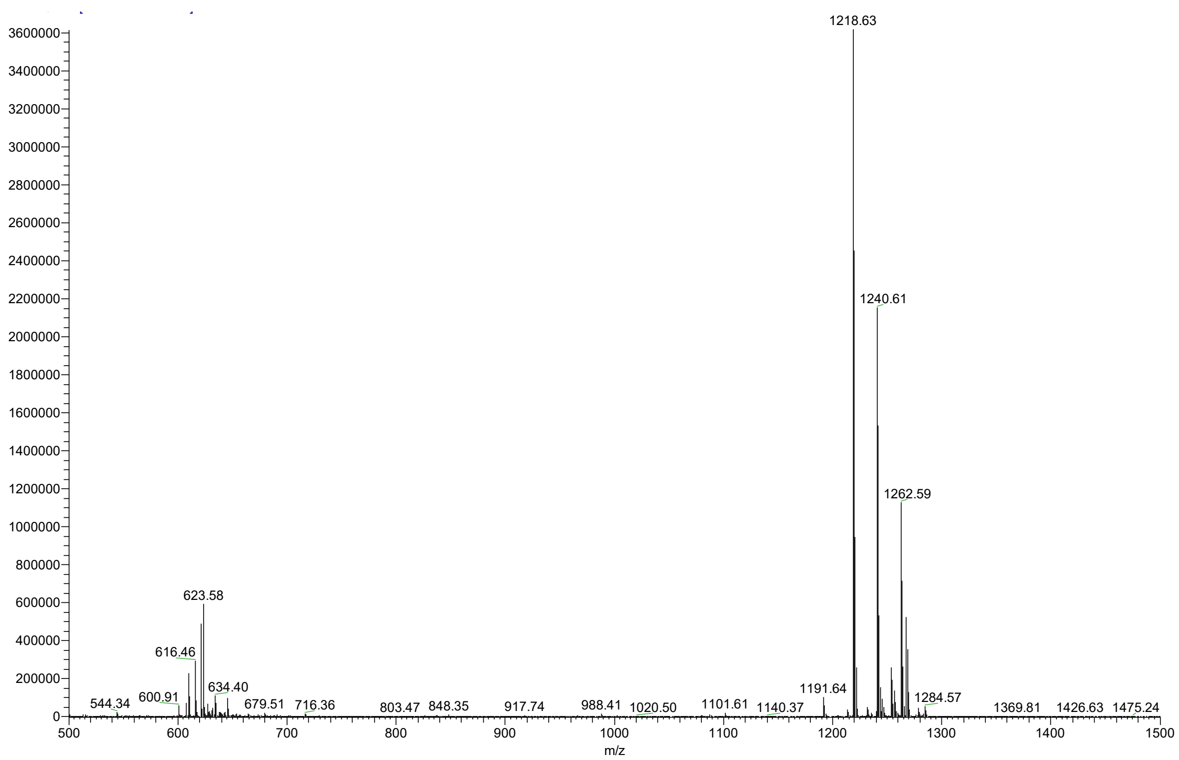

Figure S24: ESI-MS of purified teixobactin analogue **2**. Exact mass calcd. for $C_{57}H_{96}N_{13}O_{15}$ = 1218.75, found M + H^+ = 1218.63

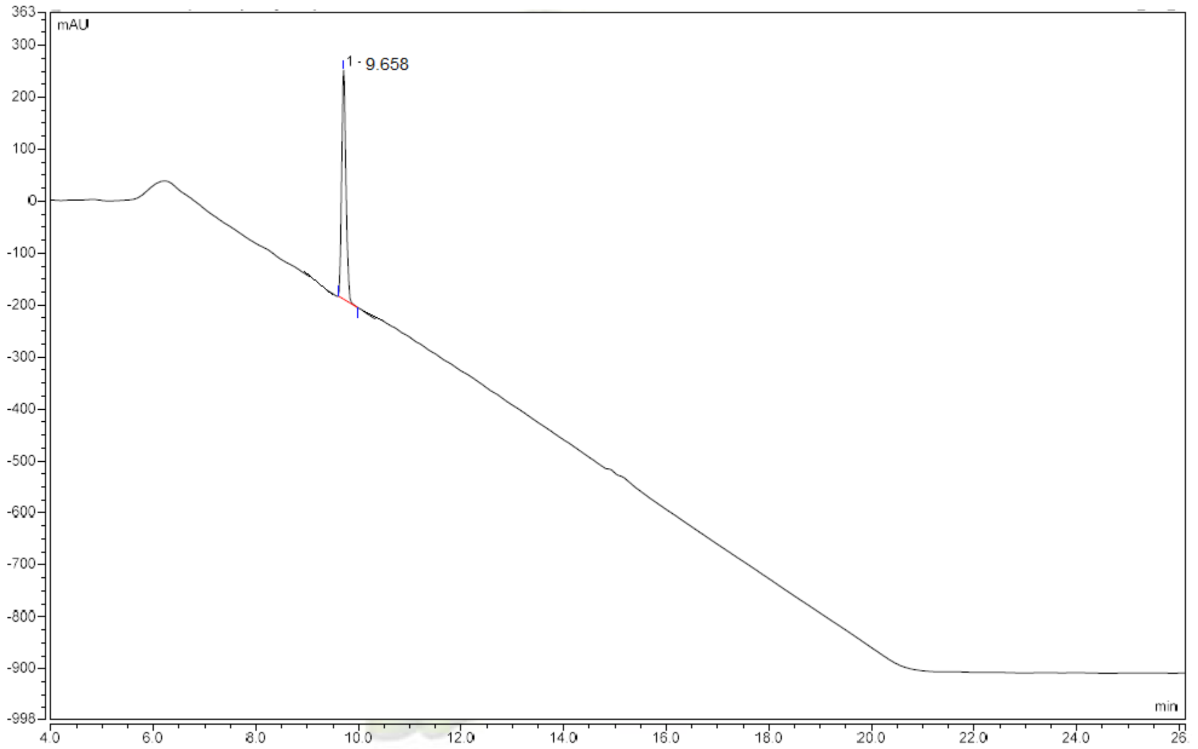

Figure S25: HPLC trace of purified teixobactin analogue **3** (gradient: 5-95% ACN in 25min using A: 0.1% HCOOH in water, B: ACN)

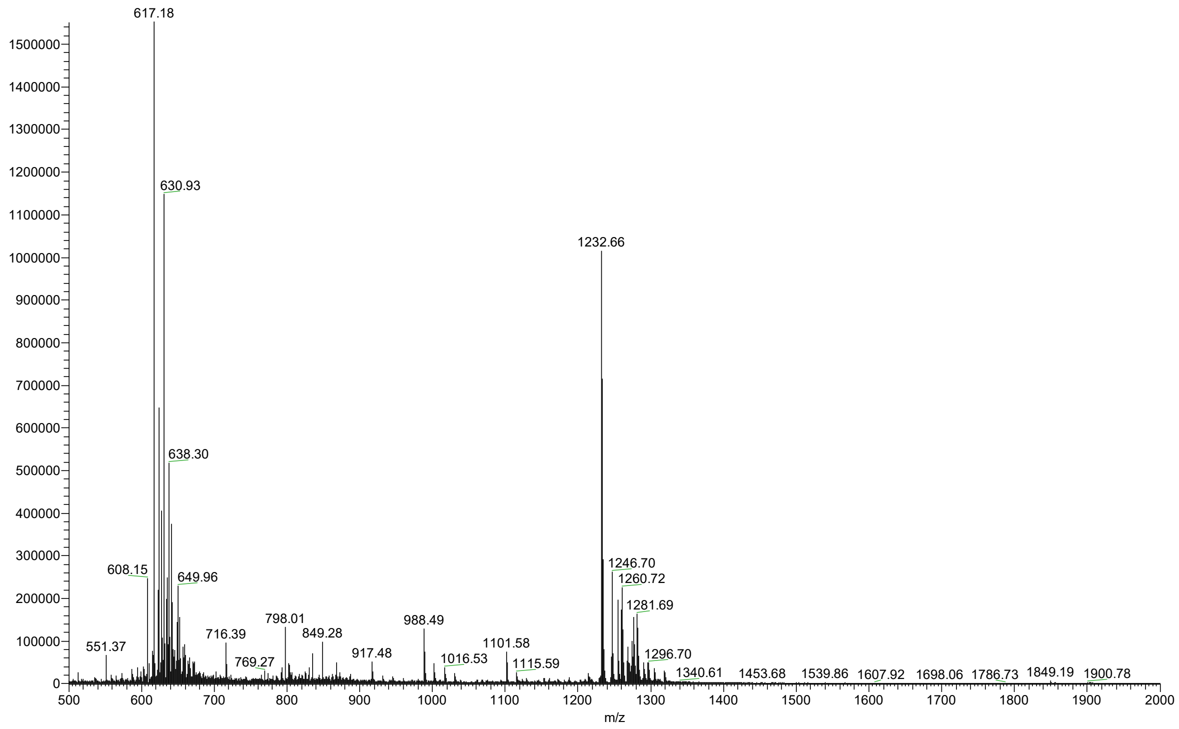

Figure S26: ESI-MS of purified teixobactin analogue **3**. Exact mass calcd. for $C_{59}H_{101}N_{13}O_{15}$ = 1232.76, found M + H^+ = 1232.66, M/2 + H^+ = 617.18

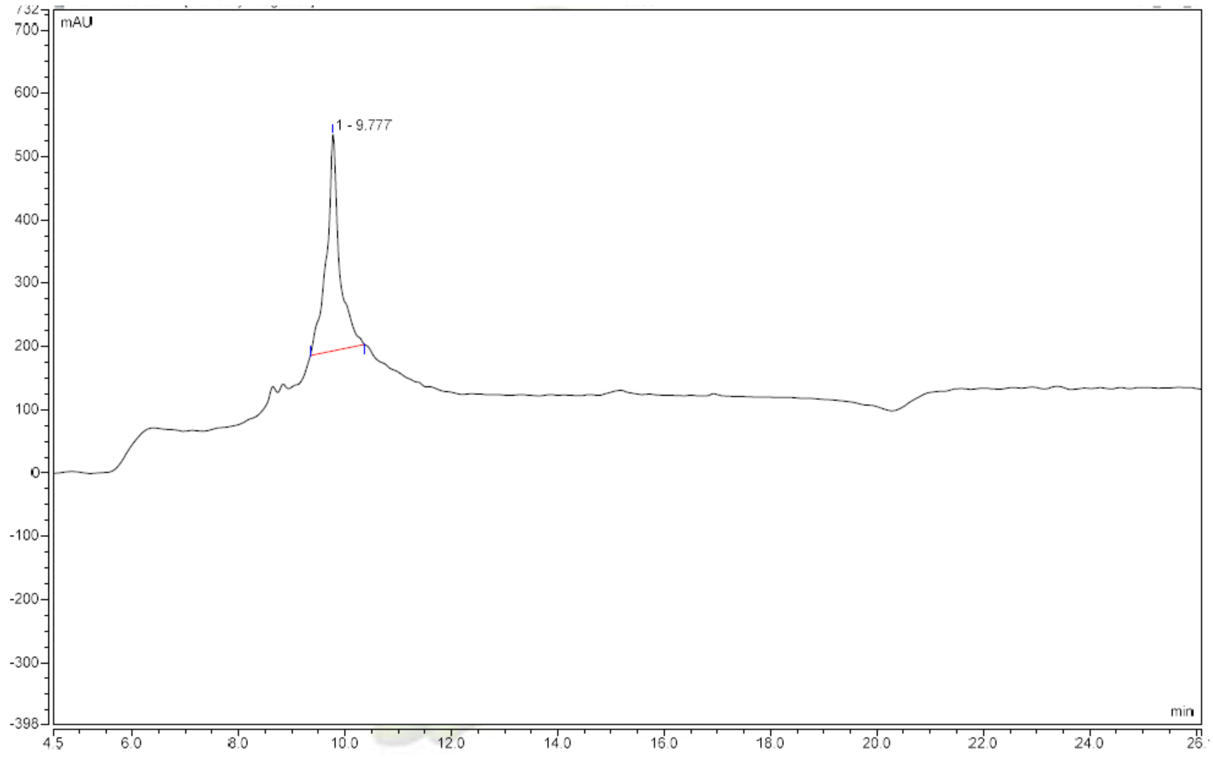

Figure S27: HPLC trace of purified teixobactin analogue **4** (gradient: 5-95% ACN in 25min using A: 0.1% HCOOH in water, B: ACN)

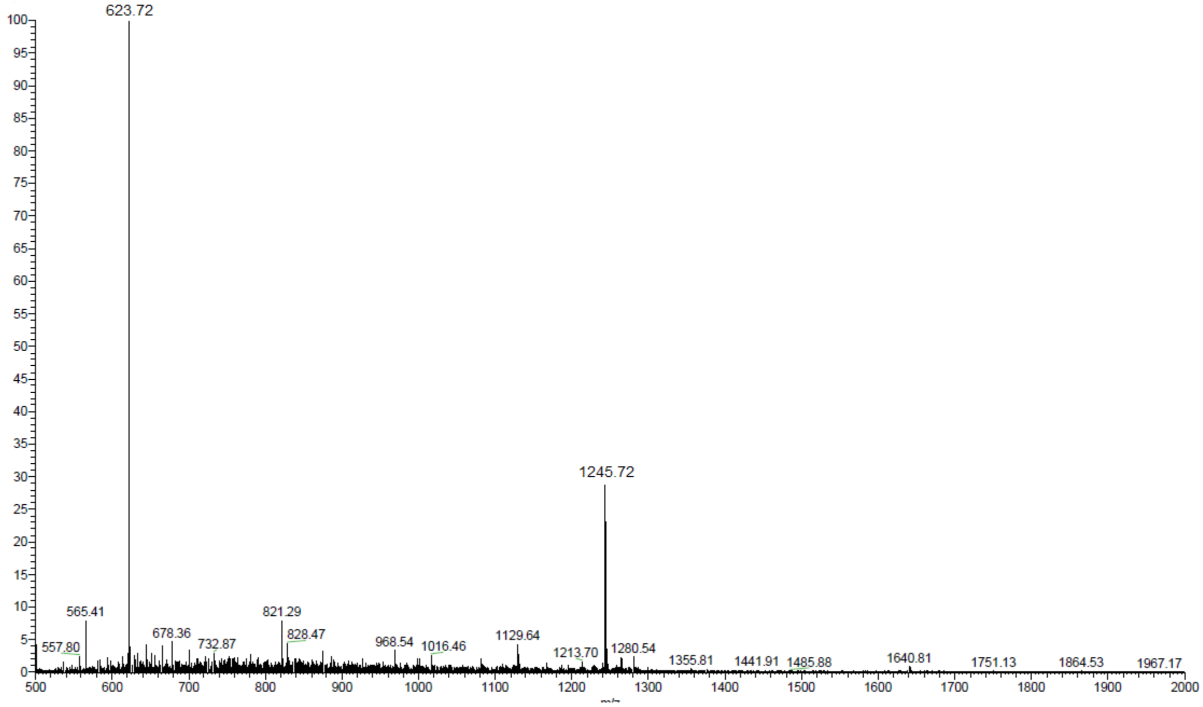

Figure S28: ESI-MS of purified teixobactin analogue **4**. Exact mass calcd. for $C_{60}H_{103}N_{13}O_{15}$ = 1246.78, found M = 1245.72, M/2 + H^+ = 623.72

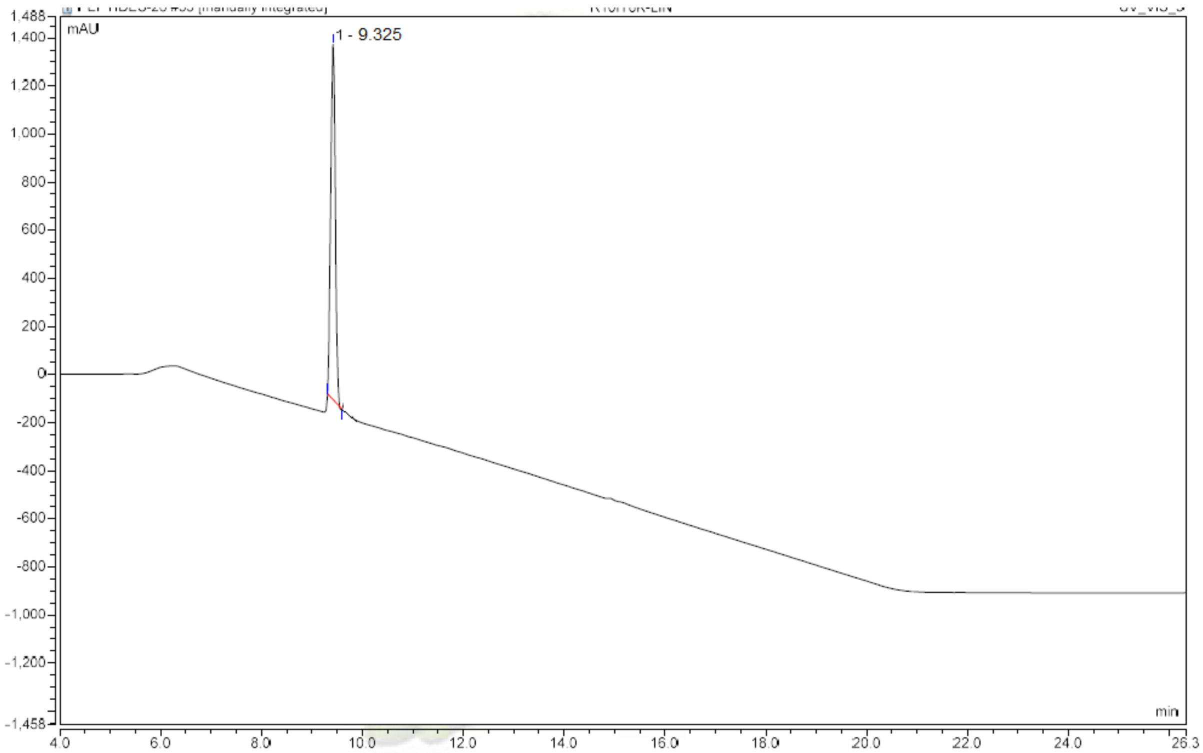

Figure S29: HPLC trace of purified teixobactin analogue **5** (gradient: 5-95% ACN in 25min using A: 0.1% HCOOH in water, B: ACN)

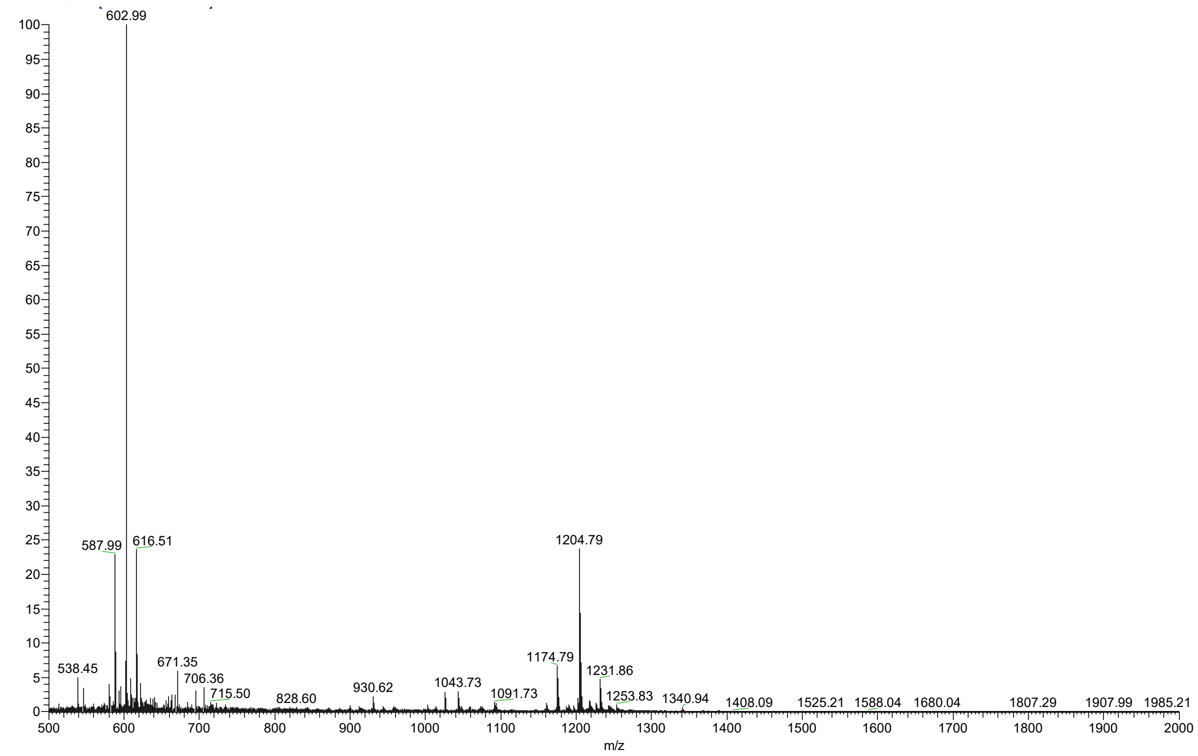

Figure S30: ESI-MS of purified teixobactin analogue **5**. Exact mass calcd. for $C_{57}H_{96}N_{12}O_{16}$ = 1205.71, found M = 1204.79, M/2 = 602.99

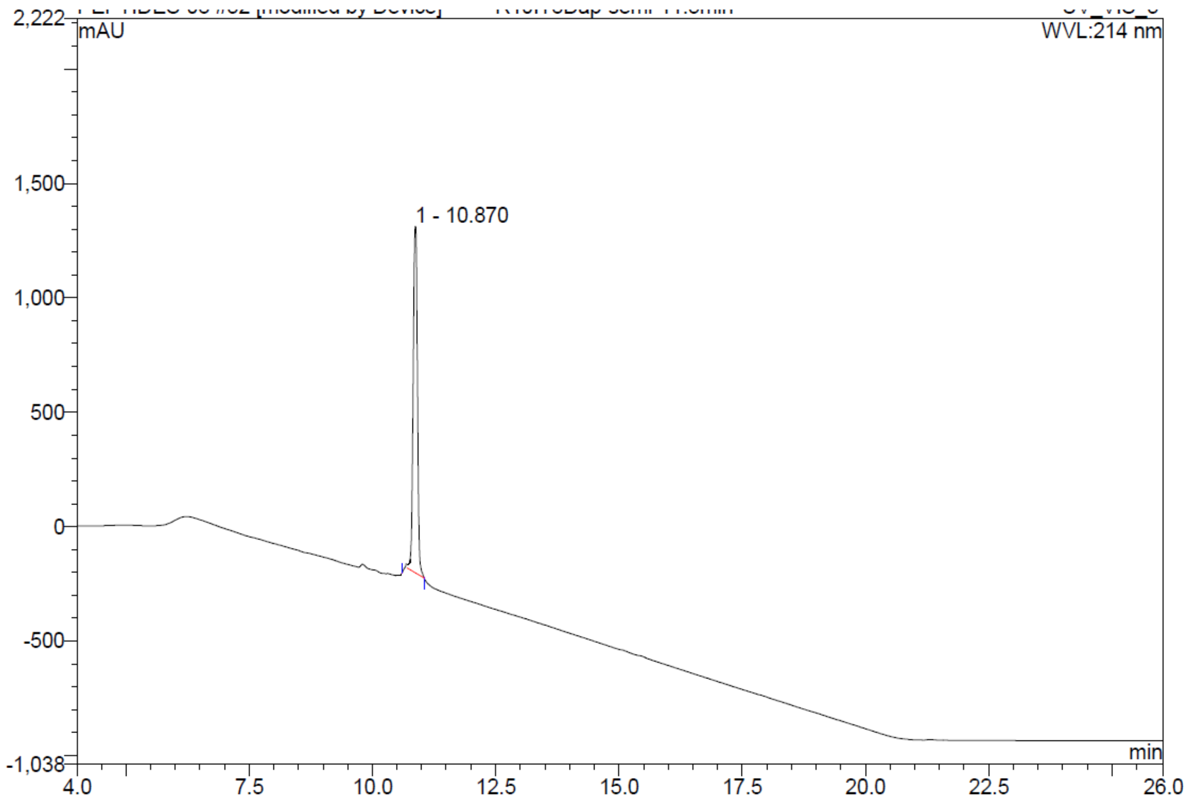

Figure S31: HPLC trace of purified teixobactin analogue **6** (gradient: 5-95% ACN in 25min using A: 0.1% HCOOH in water, B: ACN)

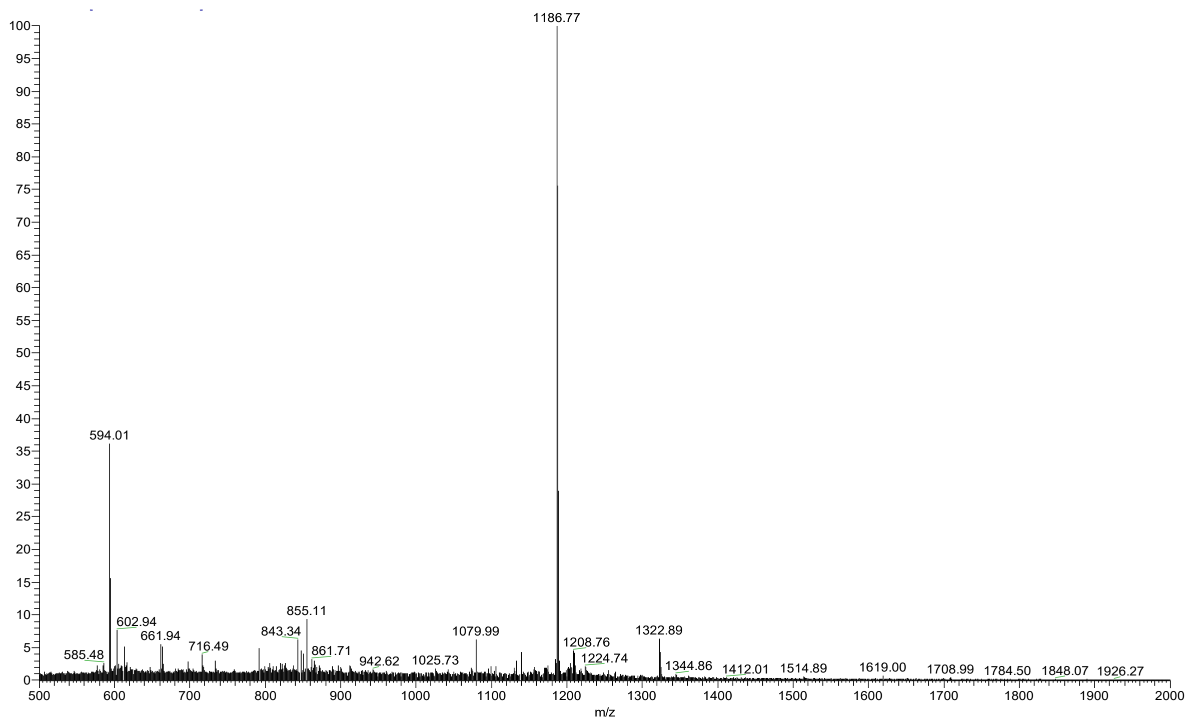

Figure S32: ESI-MS of purified teixobactin analogue **6**. Exact mass calcd. for $C_{57}H_{95}N_{13}O_{14}$ = 1186.72, found M + H^+ = 1186.77, M/2 + H^+ = 594.01

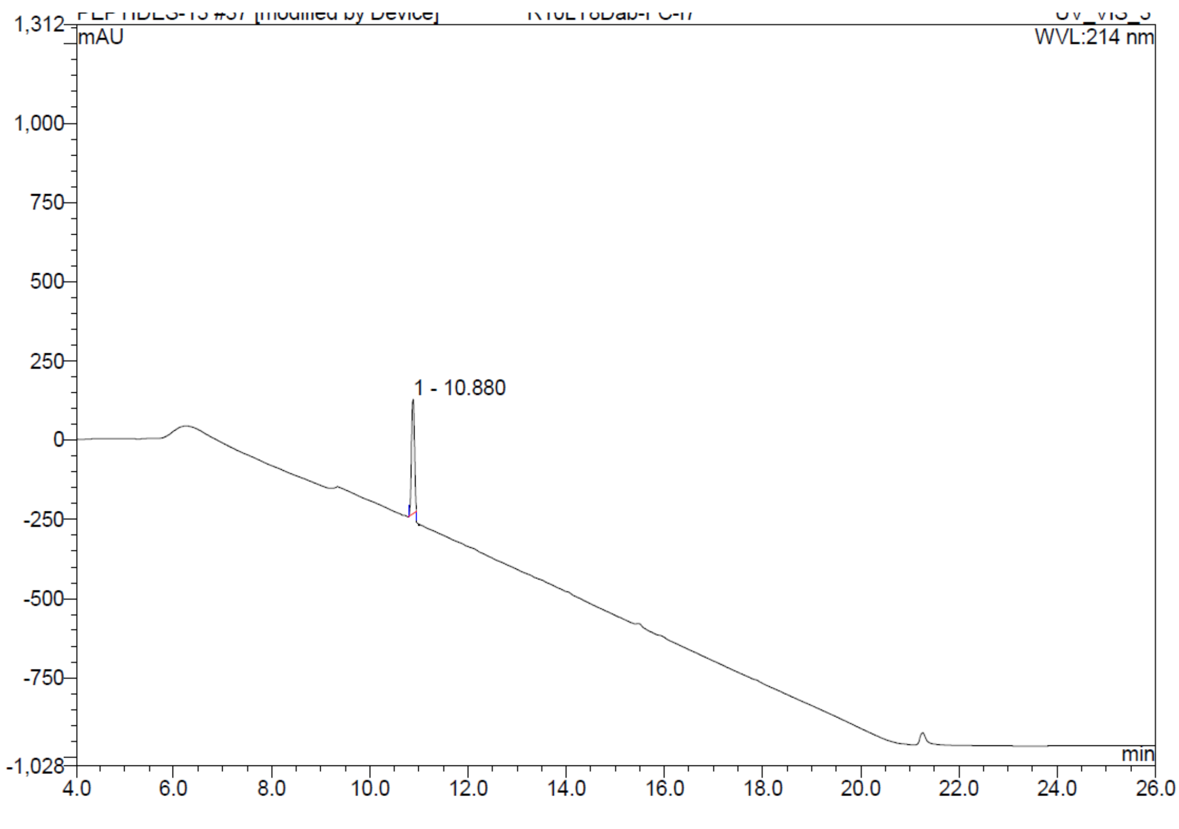

Figure S33: HPLC trace of purified teixobactin analogue **7** (gradient: 5-95% ACN in 25min using A: 0.1% HCOOH in water, B: ACN)

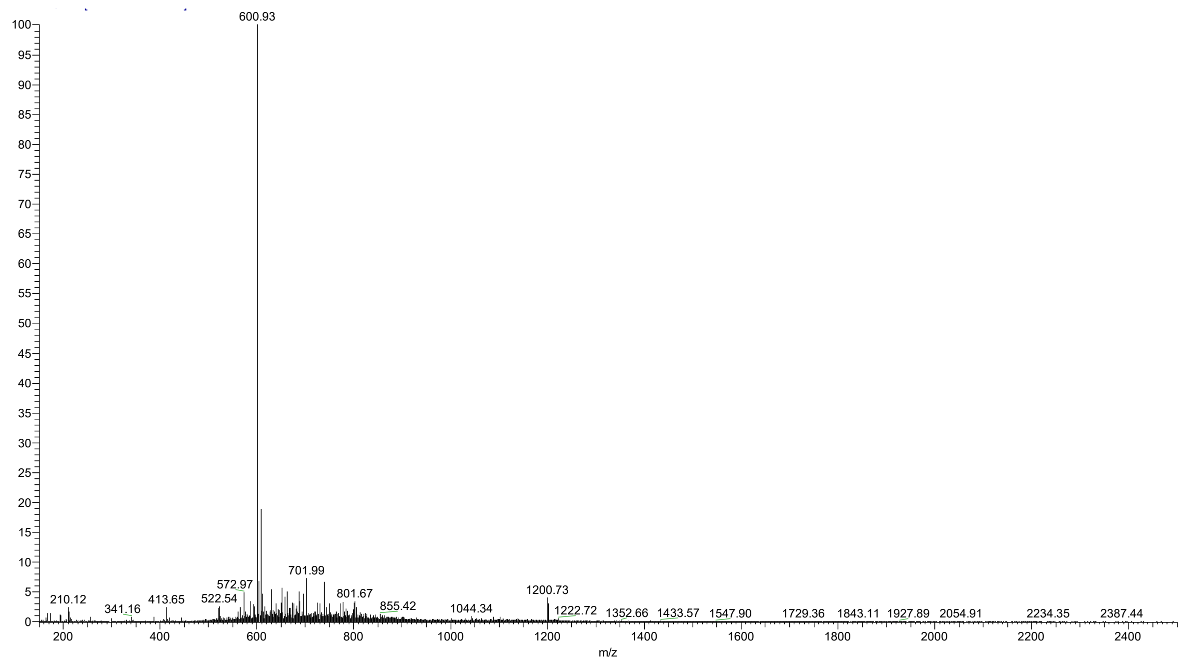

Figure S34: ESI-MS of purified teixobactin analogue **7**. Exact mass calcd. for $C_{58}H_{97}N_{13}O_{14}$ = 1200.72, found M + H^+ = 1200.73, M/2 + H^+ = 600.93

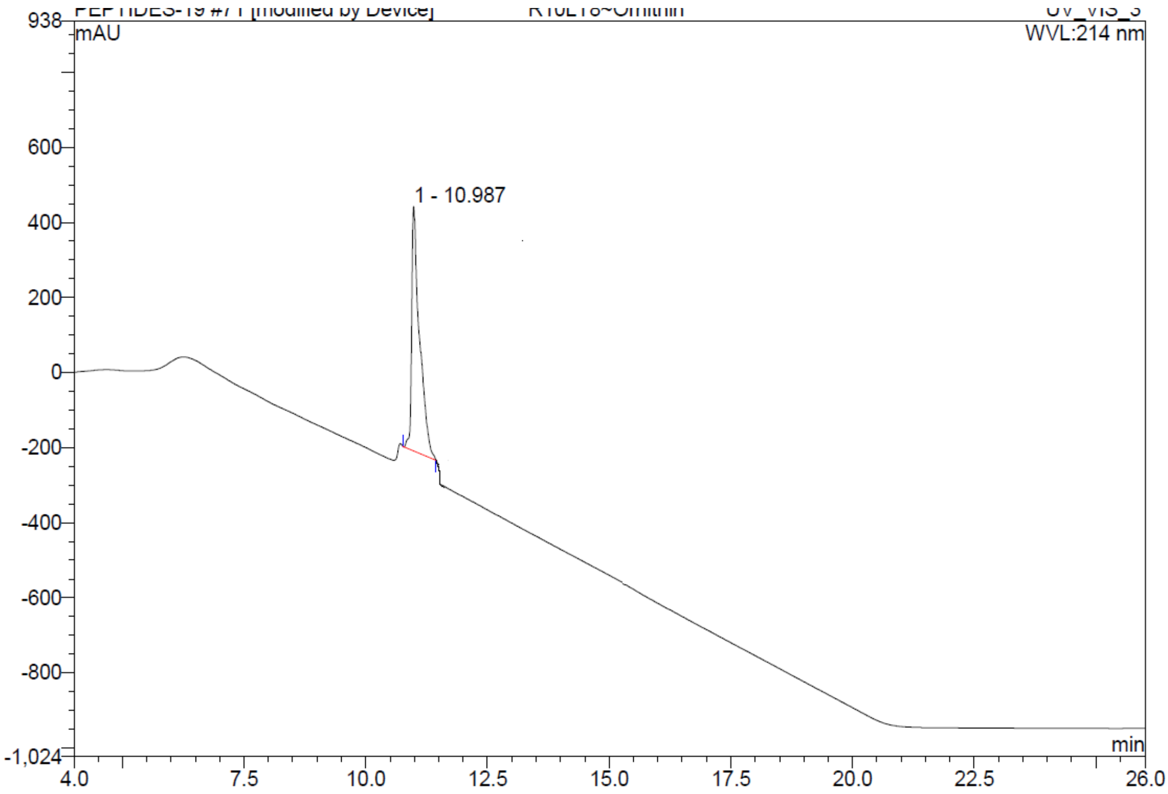

Figure S35: HPLC trace of purified teixobactin analogue **8** (gradient: 5-95% ACN in 25min using A: 0.1% HCOOH in water, B: ACN)

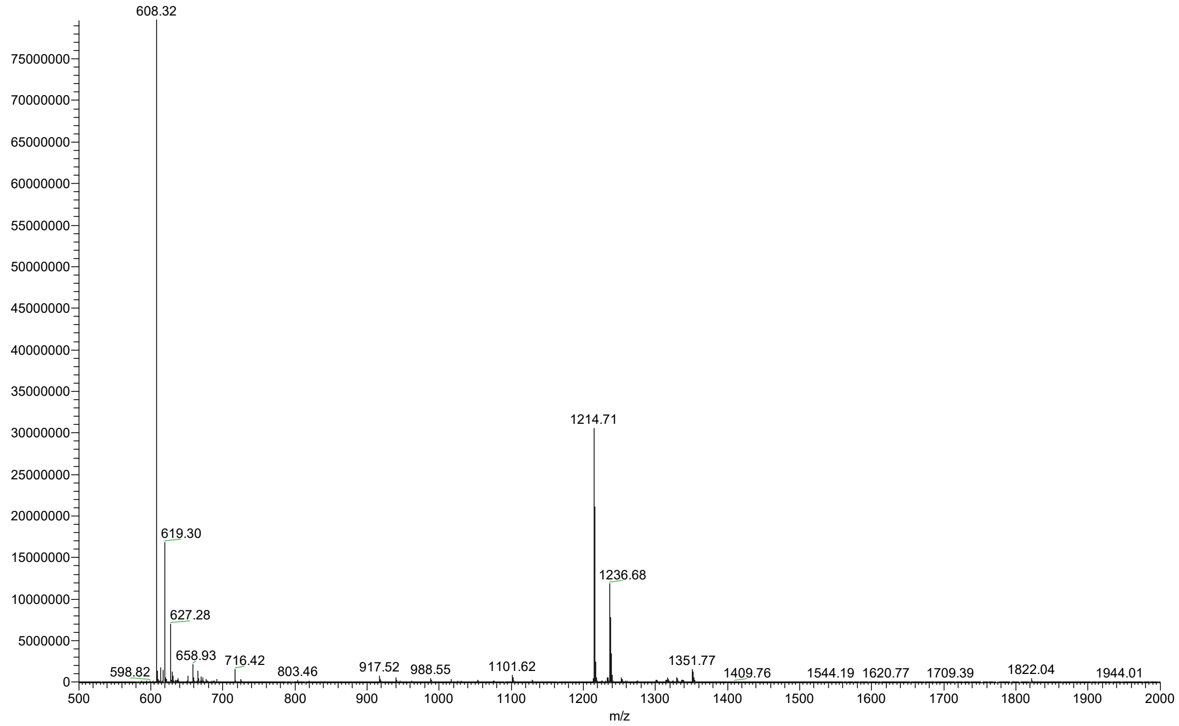

Figure S36: ESI-MS of purified teixobactin analogue **8**. Exact mass calcd. for $C_{59}H_{99}N_{13}O_{14}$ = 1214.75, found M + H^+ = 1214.71, M/2 + H^+ = 608.32

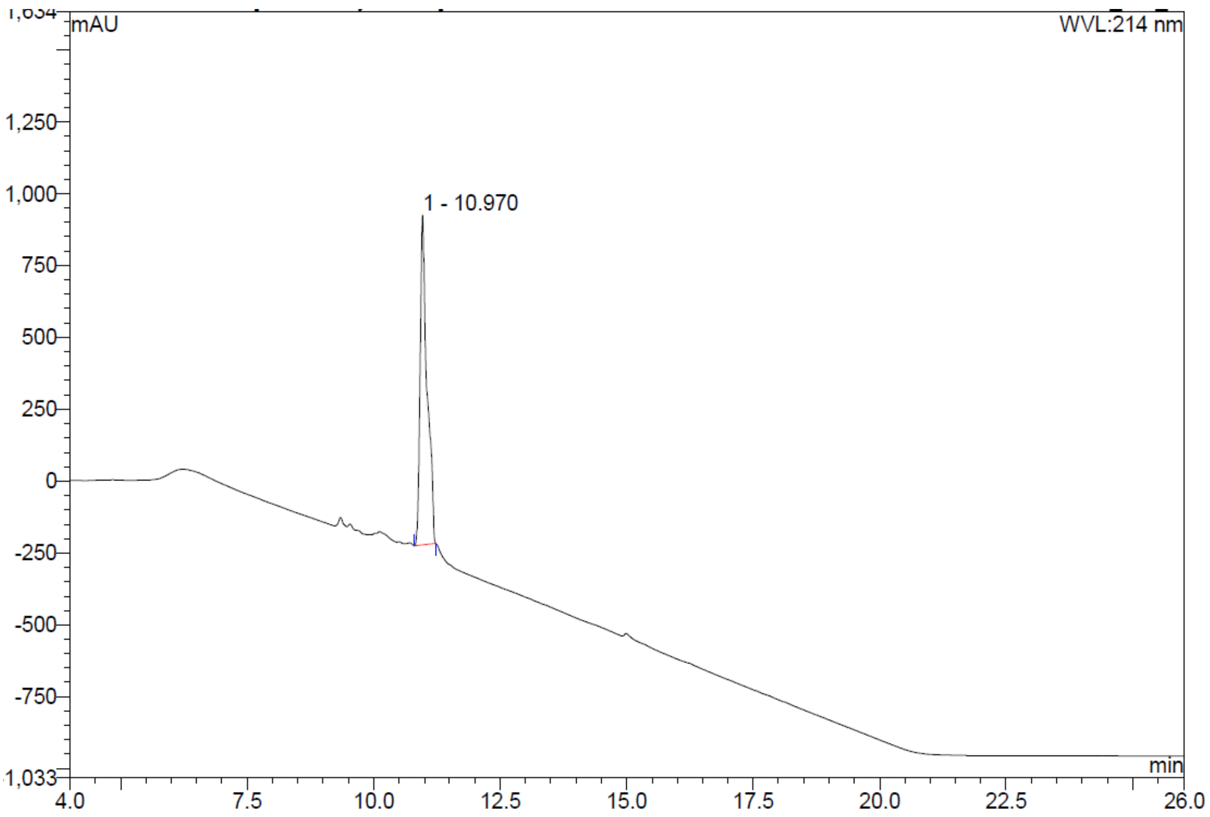

Figure S37: HPLC trace of purified teixobactin analogue **9** (gradient: 5-95% ACN in 25min using A: 0.1% HCOOH in water, B: ACN)

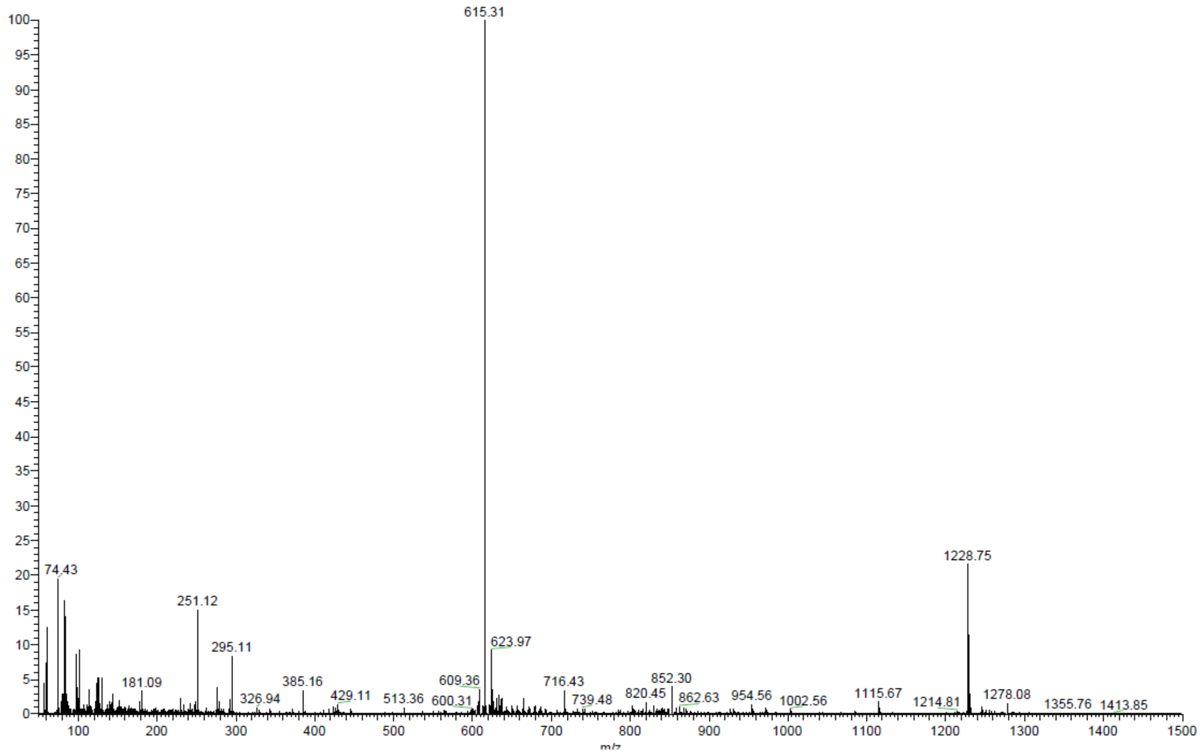

Figure S38: ESI-MS of purified teixobactin analogue **9**. Exact mass calcd. for $C_{60}H_{101}N_{13}O_{14}$ = 1228.77, found M + H^+ = 1228.75, M/2 + H^+ = 615.31

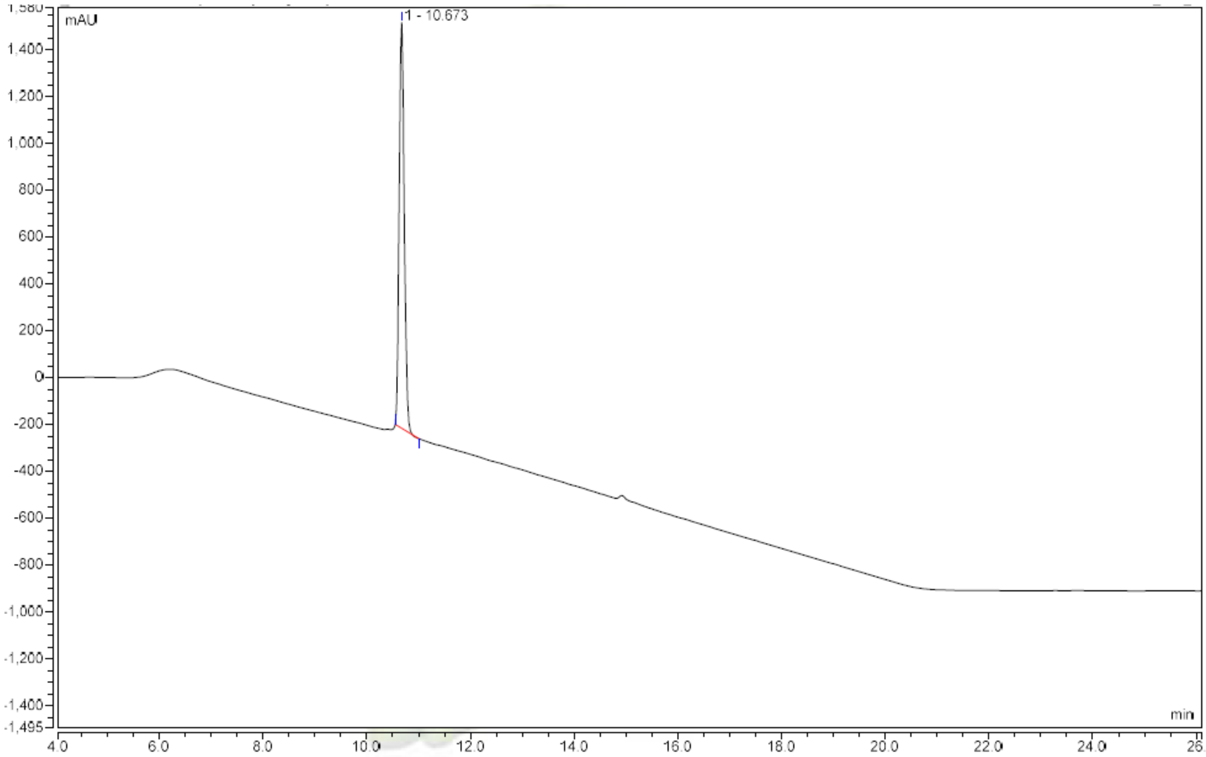

Figure S39: HPLC trace of purified teixobactin analogue **10** (gradient: 5-95% ACN in 25min using A: 0.1% HCOOH in water, B: ACN)

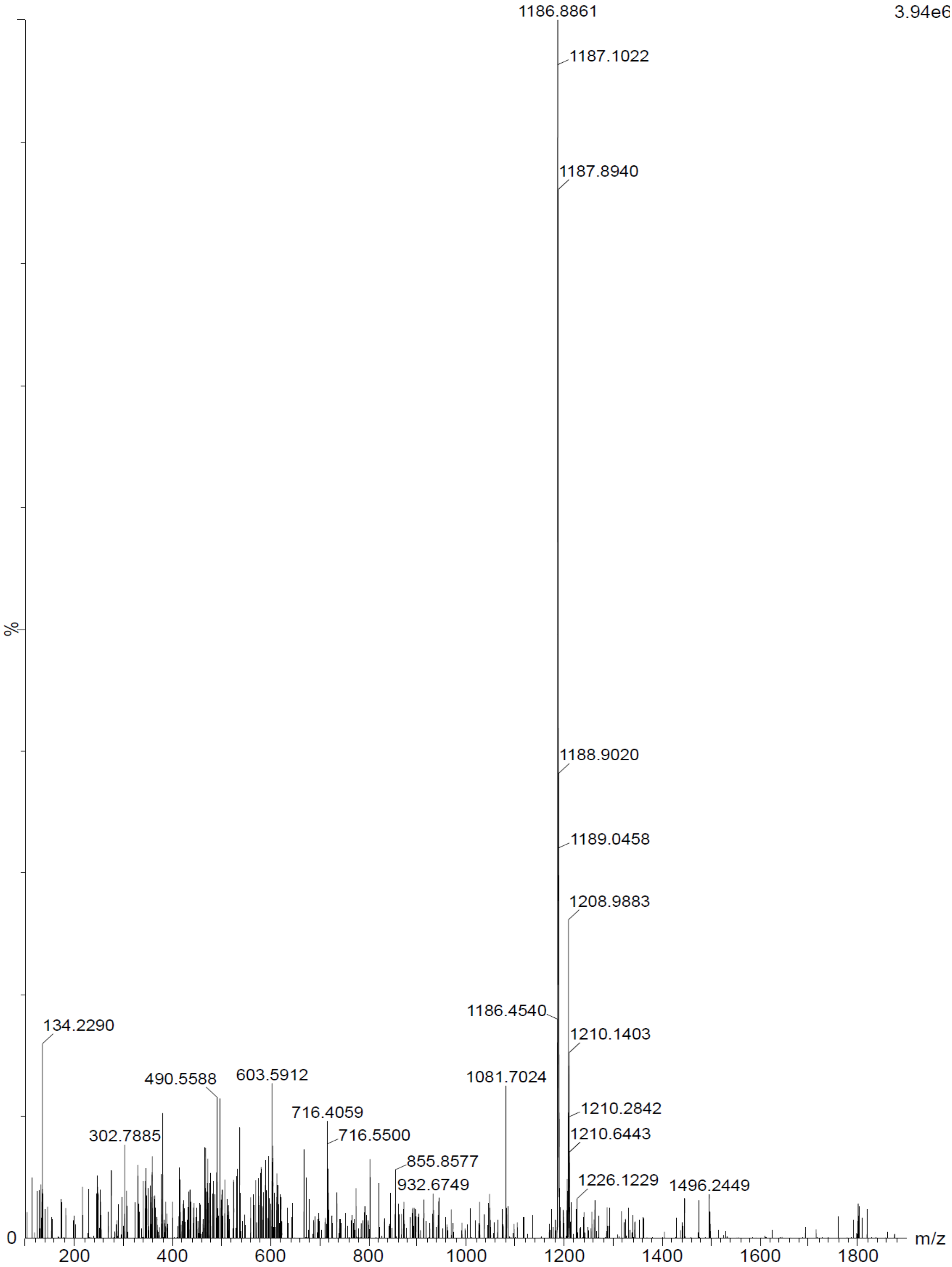

Figure S40: ESI-MS of purified teixobactin analogue **10**. Exact mass calcd. for $C_{57}H_{94}N_{12}O_{15}$ = 1187.90, found M + H^+ = 1187.10

VIII: MIC Testing (Screening)

Bacterial cultures were grown overnight in Mueller-Hinton Agar (MHA) plates and adjusted to a final concentration of 10^5–10^6 CFU/mL. 100 µL of inoculum in Mueller-Hinton broth (MHB) was mixed with an equal volume of peptides (dissolved in MHB) at 2× their concentration in a 96-well plate. In parallel experiments, MIC values were determined in media containing polysorbate 80 (0.002%, v/v) to prevent non-specific adsorption of the peptides to plastic surfaces. The final peptide concentrations ranged from 0.0625–32 µg/mL. Positive and negative controls contained 200 µL of broth with/without inoculum without peptide dissolved, respectively. The 96-well plates were incubated at 37 °C for 24 h. All experiments were performed in triplicate, and the MIC was determined as the lowest concentration at which no visible growth was observed.
